# Definition of a Family of Nonmobile Colistin Resistance (NMCR‐1) Determinants Suggests Aquatic Reservoirs for MCR‐4

**DOI:** 10.1002/advs.201900038

**Published:** 2019-04-03

**Authors:** Huimin Zhang, Wenhui Wei, Man Huang, Zeeshan Umar, Youjun Feng

**Affiliations:** ^1^ Department of Pathogen Biology & Microbiology and Department of General Intensive Care Unit of the Second Affiliated Hospital Zhejiang University School of Medicine Hangzhou Zhejiang 310058 China; ^2^ Carl R. Woese Institute for Genomic Biology University of Illinois at Urbana‐Champaign Urbana IL 61801 USA

**Keywords:** aquatic environment, colistin, lipid A, marine bacteria, MCR‐4, NMCR‐1, *Shewanella algae*

## Abstract

Polymyxins, a family of cationic antimicrobial peptides, are recognized as a last‐resort clinical option used in the treatment of lethal infections with carbapenem‐resistant pathogens. A growing body of mobile colistin resistance (MCR) determinants renders colistin ineffective in the clinical and human sectors, posing a challenge to human health and food security. However, the origin and reservoir of the MCR family enzymes is poorly understood. Herein, a new family of nonmobile colistin resistance (from *nmcr‐1* to *nmcr‐1.8*) from the aquatic bacterium *Shewanella* is reported. NMCR‐1 (541aa) displays 62.78% identity to MCR‐4. Genetic and structural analyses reveal that NMCR‐1 shares a similar catalytic mechanism and functional motifs, both of which are required for MCR action and its resultant phenotypic resistance to polymyxin. Phylogeny and domain‐swapping demonstrate that NMCR‐1 is a progenitor of MCR‐4 rather than MCR‐1/2. Additionally, the experiment of bacterial growth and viability reveals that NMCR‐1 promotes fitness cost as MCR‐1/4 does in the recipient *Escherichia coli*. In summary, the finding suggests that the aquatic bacterium *Shewanella* (and even its associated aquaculture) is a reservoir for MCR‐4 mobile colistin resistance.

## 1 Introduction

Antimicrobial resistance (AMR) has become one of the biggest threats to global health, food security, and social development.^1^ Worrisomely, O'Neill predicted that AMR crisis might cause 10 000 000 deaths per year by 2050.^2^, ^3^ The membrane‐disrupting antibiotic polymyxin is a paradigmatic member of the cationic antimicrobial cyclic peptide family, exhibiting an ability of binding to its initial target, the negatively charged lipid A moiety of lipopolysaccharides (LPS‐lipid A) anchored on outer leaflet of bacterial surface.^4^ Colistin (i.e., polymyxin E) is a last‐resort defense against carbapenem‐resistant bacterial infections. The emergence and rapid spread of a growing body of mobile colistin resistance (MCR) family enzymes is threatening the renewed interest of colistin as one of the limited options in clinic sectors.^5^, ^6^, ^7^, ^8^ To the best of our current knowledge, seven additional distinct members (from MCR‐2^9^, ^10^, ^11^, ^12^ to MCR‐8^13^) have been assigned to the MCR family in the past 2 years since the first identification of its prototype member MCR‐1 in 2016, in China.^14^ It seems likely that MCR‐1 is most prevalent in that it has been detected in nearly 50 countries covering six of seven continents.^7^, ^8^, ^15^ Occasionally, *mcr‐1* is detected on bacterial genome, hinting a relic of recombination/integration between plasmids and chromosomes.^16^, ^17^, ^18^, ^19^ The predominant pattern of *mcr‐1* transmission/spread is mediated by diversified plasmids of over ten distinct replication incompatibilities^20^ across more than ten various species (like *Escherichia coli* (*E. coli*) and *Klebsiella pneumoniae*) of *Enterobacteriaceae*.^7^, ^14^, ^21^, ^22^, ^23^, ^24^ Similar to the scenario seen with *mcr‐1* featuring with complexity in genetic heterogeneity,^7^, ^8^, ^25^ *mcr‐3* also displays the unprecedented diversity comprising more than ten genetic variants [from *mcr‐3.1* 26, 27 to *mcr‐3.12* 28]. Therefore, we favor to believe that the two major lineages of MCR family (MCR‐1 and MCR‐3) are undergoing some evolutionary selection unknown thus far. Unlike the other five rare MCR types (MCR‐2,^10^ MCR‐5,^29^ MCR‐6,^30^ MCR‐7,^31^ and MCR‐8^13^), the *mcr‐4* gives appreciably medium level of genetic heterogeneity that includes *mcr‐4.1*,^32^, ^33^ *mcr‐4.2* [Q331R],^34^, ^35^ *mcr‐4.3* [renamed from a duplicated *mcr‐4.2* (V179G & V236F)],^36^, ^37^ *mcr‐4.4* [H205N & Q331R],^34^ *mcr‐4.5* [P110L & Q331R],^34^ and *mcr‐4.6* (V236F).^38^ Evidently, the rapidly evolving MCR family augments the complexity and diversity of rendering colistin ineffective in clinical use.

**Figure 1:**
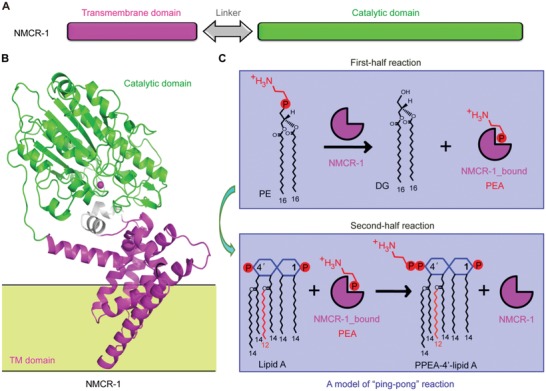
Scheme for NMCR‐1 action. A) Schematic description of the two functional motifs in NMCR‐1. B) Ribbon illustration of modeled structure of full‐length NMCR‐1. C) A putative “ping‐pong” reaction mechanism by which NMCR‐1 mediates the transfer of PEA from PE to lipid A. Linker (A) here denotes a region comprising a flexible loop between TM domain and catalytic domain. NMCR‐1_bound PEA (C) refers to an adduct of the enzyme NMCR‐1 complexed with PEA. The structural modeling is proceeded via SWISS‐MODEL, and the ribbon structure is given with PyMol. Adapted with permission.^40^ Copyright 2018, Science China Press.

Despite the unexpectedly phylogenetic diversity, all the MCR members are consistently classified into the lipid A phosphoethanolamine (PEA) transferases, a domain of the YhjW/YjdB/YijP superfamily. The neisserial EptA (renamed from LptA) is a representative member of lipid A PEA transferases,^39^ which is structurally consisted of two unique domains (transmembrane domain and catalytic motif; **Figure** **1**A,B).^41^, ^42^ Along with other research groups,^9^, ^43^, ^44^ our recent studies have partially elucidate that both EptA and MCR‐1/2 present paralleled structural/genetic requirement for enzymatic action^11^, ^45^ and its resultant phenotype,^12^, ^23^ suggesting an evolutionarily conserved mechanism for intrinsic and mobile polymyxin resistance.^11^ In particular, the biochemical data indicate that MCR‐1/2^11^, ^45^, MCR‐3,^27^ and MCR‐4^46^ consistently share a “ping‐pong” reaction machinery in the transfer of PEA to the suggestive 4′‐phosphate position of lipid A moiety via an adduct of MCR1/2/3_bound PEA, in general agreement with that of EptA.^41^, ^42^ It might underscore functional unification across MCR family enzymes (Figure 1C). Because antibiotic killing mediated by colistin is believed to connect with the reactive oxygen species (ROS) centering hydroxyl radical death pathway,^47^, ^48^ it is in rational that ROS quenching is observed in the given bacterial cells expressing either *mcr‐1/2* (and/or the progenitor *icr‐Mo* 49) or *mcr‐3*.^27^ We favor to believe that surface remodeling by MCR‐like enzymes formulates a barrier of preventing the efficient entry of colistin, and thereafter shuts off the signaling of colistin‐induced formation of exogenous ROS. Driven by a bioinformatics‐based proposal that *Moraxella* species function as possible reservoirs for MCR‐1/2 members,^50^, ^51^, ^52^ we have functionally defined a gene of *Moraxella osloensis* (AXE82_07515, referred to *icr‐Mo*), a progenitor for MCR‐1/2,^49^ whose biochemical action is validated by X‐ray structure of its catalytic domain.^53^ However, a progenitor (and/or natural reservoir) assigned to the rest of MCR members placed in phylogenetic distance from MCR‐1/2 remains a mystery.

Very recently, genomic sequencing suggests that a putative determinant of colistin resistance (*eptA*, so‐called *pmrC*)^54^, ^55^ is present in a human isolate *Shewanella algae* (*S. algae*) MARS 14, and an MDR‐*S. algae* isolates from shellfish.^56^ However, biochemical demonstration is further required. In this study, we attempt to close the missing gap of knowledge. We thereafter rename this *eptA* of *S. algae* as nonmobile colistin resistance (*nmcr‐1*). Moreover, we propose the NMCR family containing eight variants (from *nmcr‐1*, *nmcr‐1.2*, to *nmcr‐1.8*) that are restricted to certain species of *Shewanellaceae*. Among the eight members of MCR family, MCR‐4 is mostly close to NMCR‐1 (541aa) with 62.78% protein identity. As expected, NMCR‐1 shares almost identical mechanistic repertoires critical for its action and phenotypic resistance as a given MCR member does (Figure 1), and acts as a progenitor of MCR‐4 variants rather than MCR‐1/2 enzymes. Collectively, this finding demonstrates that the aquatic bacterium *Shewanella* is a reservoir for MCR‐4 variants and underlines that aquaculture should be introduced as an environmental factor into the stewardship of “One Health” (human sector, animal sector, and previously neglected environmental sector) for the maintenance of colistin as an effective option in clinical settings.^5^, ^57^, ^58^

## 2 Results

### 2.1 NMCR‐1, a Family of Nonmobile Colistin Resistance Determinants

**Figure 2:**
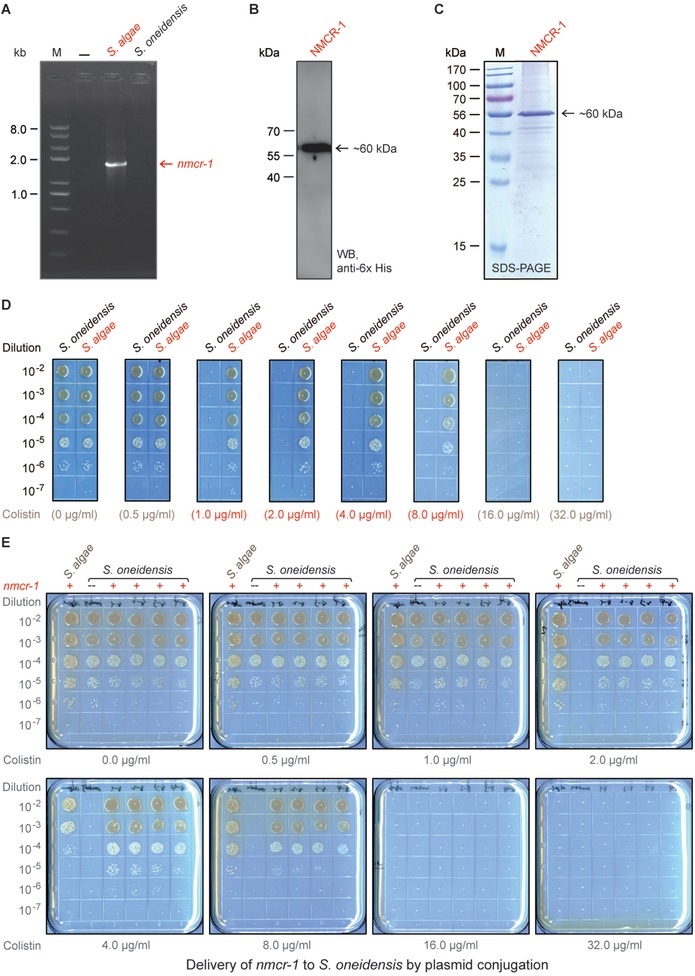
Functional definition of a novel nonmobile colistin resistance (NMCR‐1) determinant from *Shewanella algae*. A) PCR assay of *nmcr‐1* in *S. algae* and *S. oneidensis*. B) Western blot‐based analyses for expression of NMCR‐1 in *S. algae*. C) SDS‐PAGE (15%) profile of the purified NMCR‐1 protein. D) Comparative analyses of colistin resistance in *S. algae* and *S*. *oneidensis*. E) Expression of *nmcr‐1* allows the colistin‐susceptible *S. oneidensis* to grow on the nonpermissive condition supplemented with up to 8 µg mL^−1^ of colistin. Bacterial strains were kept on LBA plates with different levels of colistin. A representative result is given from over three independent experiments.

Though comparative genomics recently proposed that the locus SO_2048 (541aa) of *S. algae* encodes a putative PEA‐lipid A transferase (LptA, also called EptA),^54^, ^56^ a complete set of genetic and biochemical evidence is lacking. Indeed, we notice that *Shewanella oneidensis (S. oneidensis)* MR‐1 retains a *lptA* locus [SO_2048, 541aa] neighboring with SO_2047 (S9 protein of prolyl oligo‐peptidase family) at the 5′‐end, and SO_2049 (diguanylate cyclase) at the 3′‐end. Despite that it is annotated as a sulfatase with the domain of unknown function (DUF1705), belonging to the pfam08019 family, the *S. oneidensis* LptA has only 66.5% identity to the counterpart in *S. algae* MARS14. This somewhat gives a hint that bacterial colistin susceptibility varies between *S*. *oneidensis* and *S. algae*. As suggested by the nomenclature committee in National Center for Biotechnology Information (NCBI), we thereafter renamed the *lptA* of *S. algae* MARS14 into *nmcr‐1*, an abbreviation for nonmobile colistin resistance determinant. Using the *nmcr‐1* sequence (nucleic acids and amino acids) as a probe, we searched the sequence database of all the known genomes (and/or contigs) within the genus *Shewanella*. As a result, it returned significant hits of over 94% identity in seven additional species/strains (such as *Shewanella upenei* (*S. upenei*) and*Shewanella indica* (*S. indica*) of *Shewanellaceae*; Figures S1 and S2, Supporting Information). These heterogenic variants of *nmcr‐1* are assigned to be *nmcr‐1.2*, *nmcr‐1.3*, …, and *nmcr‐1.8*, respectively (Figure S1, Supporting Information). Indeed, polymerase chain reaction (PCR) detection with a pair of specific *nmcr‐1* primers (Table S1, Supporting Information) demonstrated that *nmcr‐1* is present in *S. algae*, but not *S. oneidensis* (**Figure** **2**A). An efficient expression of *nmcr‐1* in *E. coli* was visualized with western blot (Figure 2B) and protein purification separation (Figure 2C). This explained why *S. algae* gives appreciable growth on the Luria‐Bertani Agar (LBA) plates with up to 8.0 µg mL^−1^ colistin, whereas *S. oneidensis* only appears on the condition of no more than 0.5–1.0 µg mL^−1^ colistin (Figure 2D,E). Also, the phenotypic resistance to colistin was seen in *S. indica* having *nmcr‐1.6* (Figure S3, Supporting Information).

**Figure 3:**
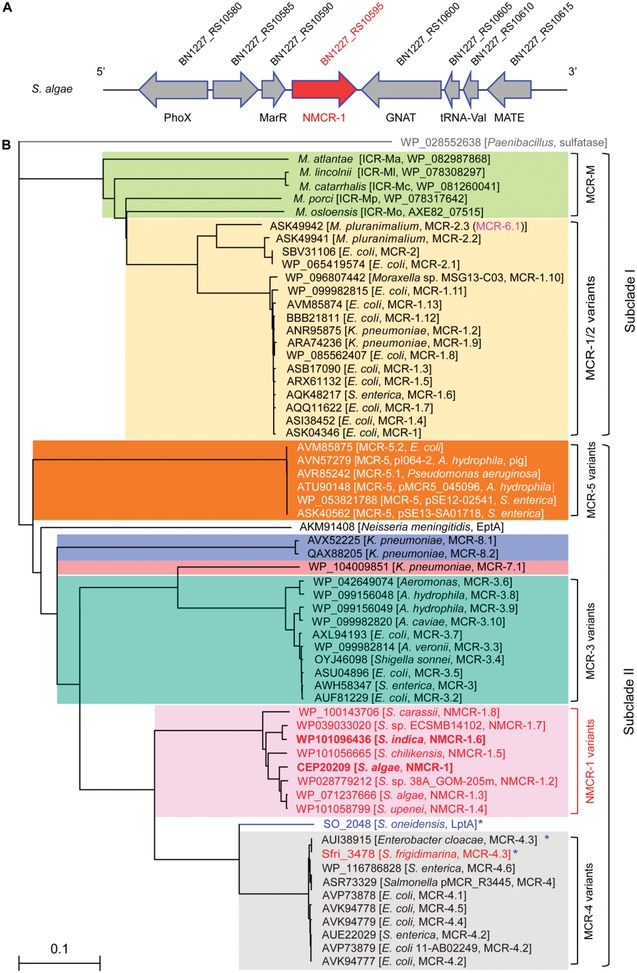
Phylogeny of NMCR‐1. A) Genetic organization of *nmcr‐1* and its neighboring loci. B) Phylogenetic analyses of NMCR‐1 and its relatives. The diversified members of MCR family were sampled from NCBI, and then were subjected to multiple sequence alignment using Clustal Omega. The resultant output of phylogenetic tree was given with TreeView. * denotes a nonfunctional PEA‐lipid A member. Among them, MCR‐4.3 (renamed from a duplicated MCR‐4.2^37^) is an inactive variant of MCR‐4 in which only two substitutions (V179G and V236F) and the LptA of *S. oneidensis* are shown here to have no ability of conferring colistin resistance (Figure 2, and Figures S1 and S3, Supporting Information). Of note, this inactive version is present on the chromosome of *S. frigidimarina*, a marine bacterium.

Genomic context of these *nmcr‐1* variants is found to be extremely conserved because its upstream gene is *marR*, encoding the MarR family of transcriptional regulator, and the downstream locus on the opposite strand encodes a bifunctional acyl‐CoA synthetase/GNAT family N‐acetyltransferase (**Figure** **3**A and Figure S2, Supporting Information). The fact that the genetic environment of *lptA* in the colistin‐susceptible *S. oneidensis* is different from the counterparts in other *Shewanella* species prompted us to examine what consequence might occur once the introduction of *nmcr‐1* into *S. oneidensis* (Figure 2D). Prior to the assays of conjugation, we engineered a donor strain of *E. coli* WM0364 to carry pHGE‐Ptac*::nmcr‐1*, a plasmid‐borne *nmcr‐1*. Then, the resultant donor strain FYJ1495 was subjected to conjugation with the recipient strain *S. oneidensis*, giving FYJ1494 (*S. oneidensis* with HGE‐Ptac*::nmcr‐1*; Table S1, Supporting Information). As expected, the acquisition of *nmcr‐1* renders *S. oneidensis* to gain the appreciable level of colistin resistance (≈8 µg mL^−1^), as *S. algae* does (Figure 2D). Therefore, it constitutes a functional proof that NMCR‐1 is a representative for a newly identified nonmobile colistin resistance exclusively restricted to certain species of *Shewanella*.

### 2.2 NMCR‐1 Acts as a Progenitor of MCR‐4

Given the fact the NMCR‐1 intrinsic enzyme and the MCR mobile element both present similar structural architecture (Figure 1A,B) and give comparable phenotypic resistance (Figure 2D,E and Figure S3, Supporting Information); therefore, we are interested in elucidation of the evolutionary relationship. Using maximum likelihood method, we constructed a molecular phylogeny (Figure 3B). Not only are the eight NMCR‐1 variants (from NMCR‐1 to NMCR‐1.8; Figure S2, Supporting Information) involved, all the major members of MCR lineage (from MCR‐1 to MCR‐8) are included (Figure 3B).^25^ Within the family of PEA‐lipid A transferases, the prototype refers to *Neisseria meningitidis* (*N. meningitidis*) EptA,^39^ and a recently proposed origin denotes the *Paenibacillus* sulfatase (Figure 3B).^23^

It is in rational that this phylogenetic tree is portioned into two distinct lineages (Subclade I and Subclade II). Subclade I is consisted of a dozen of MCR‐1/2 variants (e.g., MCR‐1.2 and MCR‐2.2) and its progenitors, like ICR‐Mo,^49^ exclusively encoded in *Moraxella* species^50^, ^51^, ^52^ (Figure 3B). Of note, MCR‐2.3 denotes an intrinsic determinant of *Moraxella pluranimalium*, which was recently renamed into MCR‐6.1.^25^, ^30^ In addition to the paradigmatic EptA of *N. meningitidis*, Subclade II comprises all the eight NMCR‐1 variants as well as the rest of MCR pan‐family (Figure 3B). In brief, a cluster of newly detected MCR‐like members (namely MCR‐7.1, MCR‐8.1, and MCR‐8.2) is neighbored with both MCR‐5 and MCR‐3 variants (Figure 3B). In particular, the presence of a certain chromosome‐encoded MCR‐3 in *Aeromonas* species^26^, ^59^, ^60^, ^61^ suggests the possibility for its origin of aquatic cultivation.^62^ Intriguingly, a collection of NMCR‐1 variants (restricted to certain species of *Shewanella*) has bridged the nonfunctional LptA (SO_2048, *S. oneidensis*) with a group of MCR‐4 variants (Figure 3B). Moreover, the plasmid‐borne MCR‐4.3 (formerly called MCR‐4.6 in *Enterobacter cloacae* 37) completely matches Sfri_3478 of *Shewanella frigidimarina* (*S. frigidimarina*). MCR‐4.3 is an inactive version in that it has two point‐mutations of V179G and V236F.^37^ Genomic context of Sfri_3478 reveals that it is surrounded with the upstream resolvase‐encoding gene, Sfri_3417, and the downstream locus, Sfri_3419, which encodes a Tn*5044*‐family transposase. It is a hint for horizontal transfer for *mcr‐4.3* from *S. frigidimarina* into diversified species of *Enterobacteriaceae* cohabitating in the ecosystem of aquaculture.^62^, ^63^ Because the revertant of MCR‐4.3 can partially restore its action, we therefore favor to believe that progressive evolution drives the gain of function in colistin resistance.

The fact that current situation of NMCR‐1 (the MCR‐4 homolog) in *Shewanella* is extremely similar to the scenario with MCR‐M, a MCR‐1/2 homolog (e.g., ICR‐Mo^49^) in *Moraxella*,^50^, ^51^, ^52^ allows us to anticipate that the family of *nmcr‐1* constitutes a progenitor of *mcr‐4* determinants (Figures 2 and 3 and Figure S2, Supporting Information), i.e., the aquatic bacterium *Shewanella* acts as a resource and/or reservoir for MCR‐4 colistin resistance (Figure 2D,E). Further genetic and biochemical investigation of a representative of NMCR‐1 family is needed for better understanding the ecological roles of *Shewanella* in distribution, evolution, and functional gain of *mcr‐4*.

### 2.3 Characterization of NMCR‐1 and Its Action

With the program of TMHMM (http://www.cbs.dtu.dk/services/TMHMM/), the prediction of protein topology suggested that the *S. algae* NMCR‐1 (541aa) is an integral membrane protein with five trans‐membrane (TM) helices at its N‐terminus (Figure S1, Supporting Information), which is a universal hallmark among the enzymes of MCR family.^7^ To further examine the biochemical properties of NMCR‐1, we are successful in the development of an improved expression system of pBAD24‐8xHis/BL21(pLysS) used for this integral membrane protein (Table S1, Supporting Information). It was judged with western blot (Figure 2B). As expected, we harvested the full‐length NMCR‐1 protein with C‐terminal 8xHis tag, and purified them to homogeneity (Figure 2C). Circular dichroism (CD) spectrum of NMCR‐1 displayed two unique absorbance peaks at the wavelength of 208–220 nm, typical of α‐helix‐rich protein (Figure S4A, Supporting Information). Inductively coupled plasma mass spectrometry (ICP‐MS) demonstrated that zinc is occupied by NMCR‐1 protein (Figure S4B, Supporting Information). In addition, the identity of recombinant NMCR‐1 protein with an estimated mass of 60 kDa was further confirmed by peptide‐mass fingerprinting with a coverage of 73.75% (Figure S4C, Supporting Information).

Structure modeling with the neisserial EptA (PDB: 5FGN) as a template^42^ generated the architecture of NMCR‐1 in full length (Figure S5, Supporting Information). Consistent with the interpretation of TM prediction (Figure S1A, Supporting Information) and CD spectrum (Figure S4A, Supporting Information), the NMCR‐1 is topologically comprised of two distinct domains (Figure 1A,B and Figure S5A, Supporting Information): a catalytic domain at C‐terminus is connected with TM domain at N‐terminus by a flexible linker. The N‐terminal end of this linker is attached into the innermembrane by bridge helices (BH) toward the periplasm side (Figure S5B, Supporting Information). Apart from five common TM helices (TMH1‐TMH5), the TM domain also possesses four unusual small periplasmic facing helices [PH2 and PH2′ between TMH3 and TMH4; PH3 and PH4 between TMH5 and BH] (Figure S5A, Supporting Information). On the other hand, the catalytic domain is comprised of 10 α‐helices mixed with seven β‐sheets (Figure S5A, Supporting Information), featuring with a typical fold of zinc‐bound hydrolase (Figure 1B). As recently observed with four mobile elements (MCR‐1,^45^ MCR‐2,^11^ MCR‐3,^27^ and MCR‐4^46^) and two nonmobile enzymes (EptA^41^, ^42^ and ICR‐Mo^49^), a putative nucleophilic residue, threonine at the position of 277 (T277), is positioned in close proximity of the Zn^2+^ ion‐dependent active sites of NMCR‐1 (Figure 1B, and Figures S1B and S4B, Supporting Information). Whereas, its physiological role of T277 requires experimental validation.

**Figure 4:**
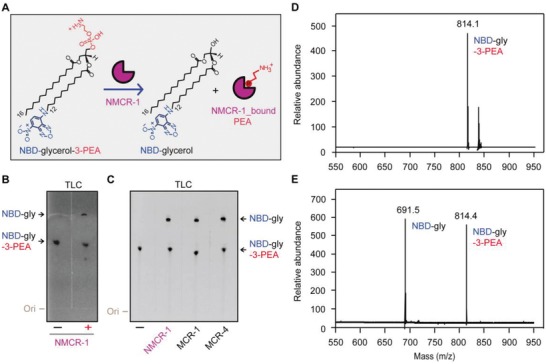
The enzymatic activity of NMCR‐1. A) Scheme for hydrolysis of an alternative substrate, NBD‐glycerol‐3‐PEA by NMCR‐1, giving NMD‐glycerol product and an adduct of NMCR‐1_bound PEA. B) TLC analyses of the hydrolysis mixture in the NMCR‐1 reaction with NBD‐glycerol‐3‐PEA as substrate. C) Parallels in the enzymatic activity of NMCR‐1 and MCR‐1/4. D) LC/MS profile of the substrate NBD‐glycerol‐3‐PEA. E) LC/MS analyses of the mixture in the NMCR‐1 reaction.

**Figure 5:**
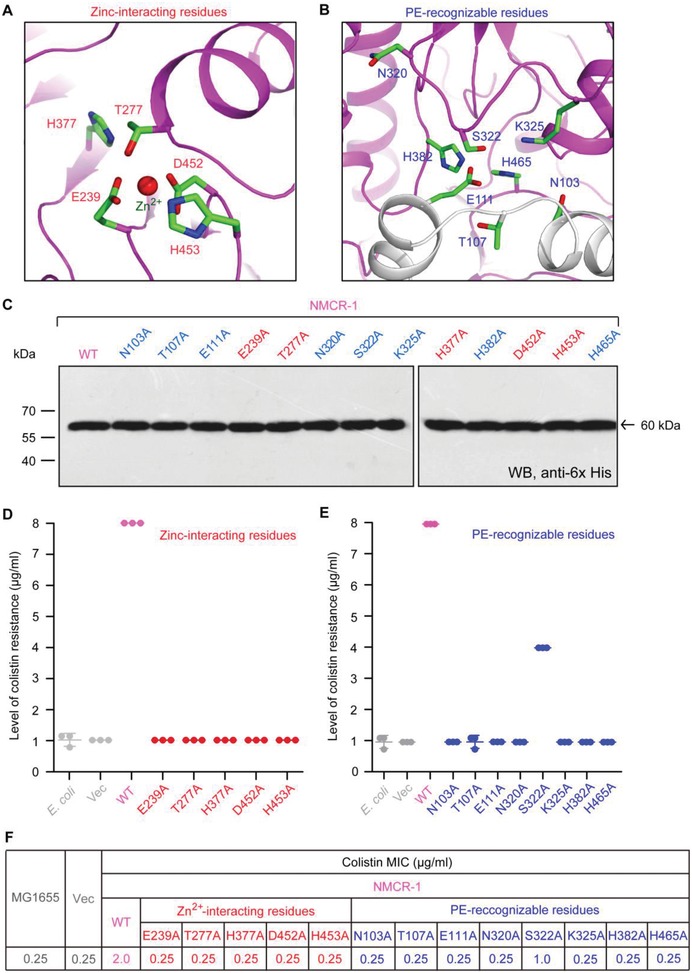
Structure and function studies of NMCR‐1 colistin resistance. A) An enlarged illustration for a five residue‐containing, Zn^2+^‐binding motif. The five residues in Zn^2+^‐binding motif of NMCR‐1 refer to E239, T277, H377, D452, and H453, respectively. B) An enlarged view of the eight residue‐containing motif involved in binding of NMCR‐1 to the PE lipid substrate. The eight residues denote N103, T107, E111, N320, S322, K325, H382, and H465, respectively. C) Use of western blotting to assay the expression of NMCR‐1 and its 13 point‐mutants in *E. coli*. D) Site‐directed mutagenesis analyses for the Zn^2+^‐binding motif of NMCR‐1 in the context of colistin resistance using the colistin susceptibility tests. E) Colistin susceptibility based dissection of the PE‐interactive residues of NMCR‐1. Three independent experiments were conducted, and each dot represents the value from in a given assay. F) Comparative analyses of NMCR‐1 and its point‐mutants in the context of colistin MIC.

**Figure 6:**
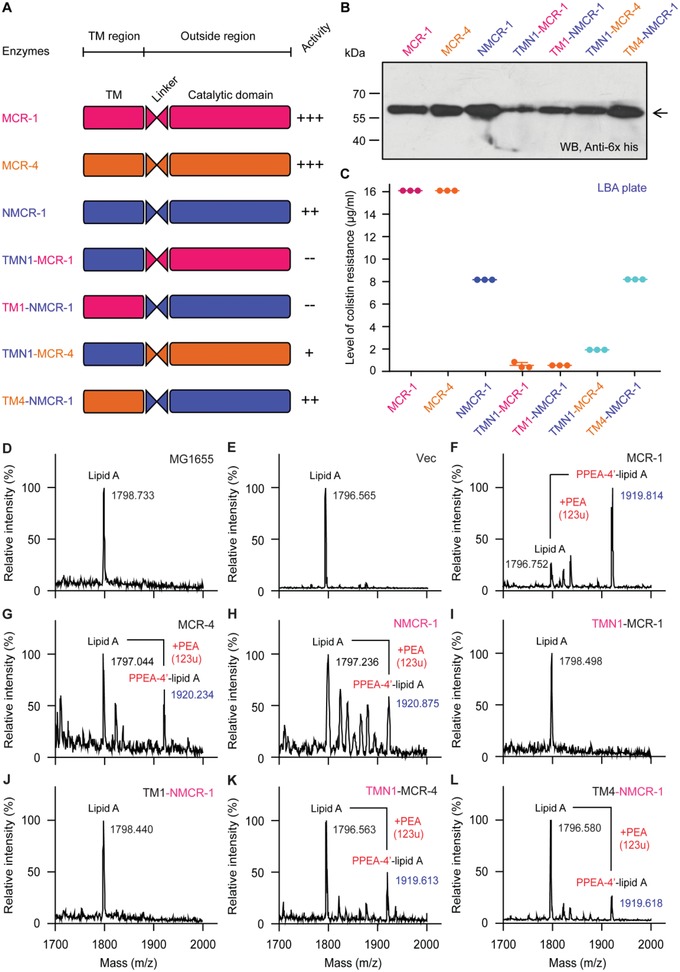
Domain‐swapping analyses of NMCR‐1 and MCR‐1/4 proteins. A) Scheme for domain‐swapped constructs between NMCR‐1 and MCR‐1/4. B) western blot assays for the expression of *nmcr‐1* and its mosaic versions in *E. coli*. C) Comparison of bacterial viability of *E. coli* expressing *nmcr‐1* and its hybrid derivatives on the LBA plates with varied level of colistin. The data are given from three independent experiments. MALDI‐TOF mass spectrometry of the LPS‐lipid A from the *E. coli* MG1655 alone D) or carrying the empty vector pBAD24 E) MS profile of the LPS‐lipid A of the *E. coli* MG1655 having F) MCR‐1 or G) MCR‐4. H) MS illustration that NMCR‐1 transfer PEA to lipid A (*m/z*, 1797.236), producing the PPEA‐4′‐lipid A (*m/z*, 1920.875). The two domain‐swapped versions (i.e., I) TMN1‐MCR‐1 and J) TM1‐NMCR‐1) between MCR‐1 and NMCR‐1/3 cannot modify lipid A in the strain MG1655. The two domain‐swapped derivatives K) TMN1‐MCR‐4 and L) TM4‐NMCR‐1) of NMCR‐1 and MCR‐4 remain functional in the transfer of PEA to the lipid A moieties.

As previously described with MCR‐1/2/3^11^, ^27^, ^45^ and ICR‐Mo,^49^ we tried molecular docking with the modeled structure of NMCR‐1 and its substrate, lipid phosphatidylethanolamine (PE) molecule. As a result, it gave a cavity positioned at the interface between TM domain and soluble domain (Figure S5B,C, Supporting Information). The measurement of this cavity suggested that it has enough space to hold the head of PE substrate (Figure S5C, Supporting Information), which was subsequently verified to participate into an interaction with zinc ion surrounded by active sites in the other cavity (Figure 5A). Evidently, this result illustrates the unexpected parallels of substrate PE‐recognizable cavity between NMCR‐1 (Figure 5B and Figure S5, Supporting Information) and MCR‐like members.^11^, ^27^, ^45^ This is reasonable to speculate that NMCR‐1 evolves a common mechanism for catalytic action shared among MCR‐like enzymes.^7^, ^27^, ^49^ Thereafter, we established an in vitro system of enzymatic reaction catalyzed by NMCR‐1 as Anandan et al.^42^ described. In this reaction, an alternative substrate for NMCR‐1 refers to a fluorescently labeled substance, NBD‐glycerol‐3‐PEA. In principle, NMCR‐1 can remove the PEA head from NBD‐glycerol‐3‐PEA, releasing the product of NBD‐glycerol and an intermediate of NMCR‐1_bound PEA (**Figure** **4**A). The incubation of MCR‐like enzyme with NBD‐glycerol‐3‐PEA at 25 °C for 24 h is a prerequisite for the subsequent separation, detection, and identification of resultant product, NBD‐glycerol (Figure 4B,E). Indeed, thin layer chromatography (TLC) revealed that the addition of NMCR‐1 leads to the generation of NBD‐glycerol product from its reactant NBD‐glycerol‐3‐PEA (Figure 4B). The in vitro action of NMCR‐1 is quite same as MCR‐1/2 does (Figure 4C). Moreover, liquid chromatography (LC) mass spectrometry confirms the identity of the reactant NBD‐glycerol‐3‐PEA (*m/z* 814.1–814.4; Figure 4D) and its resultant product NBD‐glycerol (*m/z* 691.5; Figure 4E). Subsequently, the question we might ask is where the adduct of NMCR‐1_bound PEA has been transferred. Matrix‐assisted laser desorption/ionization time‐of‐flight (MALDI‐TOF) mass spectrometry revealed that the derivative of lipid A with an addition of PEA, PEA‐4′‐lipid A, consistently appears in the *E. coli* MG1655 harboring *nmcr‐1*, whereas not in the *E. coli* alone (Figure 6D–H and Figure S7A–C, Supporting Information). Evidently, bacterial lipid A is a recipient in the transfer of PEA by NMCR‐1 via an intermediate of NMCR‐1_bound PEA (Figure 1C). Therefore, we believed that NMCR‐1 also exploits a “ping‐pong” mechanism for catalytic reaction (Figure 1C), which is required for resultant phenotypic resistance.

### 2.4 Genetic Determinants of NMCR‐1

Despite that NMCR‐1, a candidate progenitor for MCR‐4, is placed into a distinct evolutionary branch from those of both MCR‐3 and MCR‐4 (Figure 3B), extensive assays of molecular docking elucidated that a putative PE substrate‐interacting cavity is occupied within NMCR‐1 (**Figure** **5**B and Figure S4C, Supporting Information). Further analyses of modeled structure of the PE‐bound NMCR‐1 illustrated that i) a zinc‐dependent enzymatic center involves five conserved residues (namely E239, T277, H377, D452, and H453; seen in Figure 5A) and ii) eight residues (namely N103, T107, E111, N320, S322, K325, H382, and H465) might contribute to the formation of such PE‐recognizable cavity (Figure 5B). It seems very true that this PE cavity converges to the zinc‐dependent catalytic center (Figure 5A), which is almost same as MCR‐1/2,^11^, ^45^ MCR‐3/4,^27^, ^46^ and/or ICR‐Mo^49^ does. As such, structural and functional unification of both PE cavity and the active site pocket is presumed across the whole family of MCR‐like enzymes.^46^

A plasmid‐borne *nmcr‐1* was subjected to alanine substitution of all the aforementioned residues. As a result, all the 13 point‐mutants of NMCR‐1 were systematically judged in vitro and in vivo. First, the experiment of western blotting verified that all the *nmcr‐1* mutant can express as well as its parental version (Figure 5C), which constitutes a prerequisite for us to follow up their role in the phenotypic resistance to polymyxin. As anticipated, any mutation to the above five active sites impairs its ability of conferring the appearance of recipient strain *E. coli* MG1655 on the nonpermissive growth condition (i.e., LBA plates with up to 8 µg mL^−1^ colistin; Figure 5D). Except that the S322A mutant of NMCR‐1 retains partial activity allowing MG1655 to appear on the LBA plates with ≤4 µg mL^−1^ colistin (Figure 5E), the rest of the eight mutants with deficiency in PE cavity completely cannot render MG1655 to grow on the LBA plates with over 1 µg mL^−1^ colistin (Figure 5E). In general consistency with the results of bacterial viability (Figure 5D,E), the measured value of colistin minimum inhibitory concentration (MIC) (µg mL^−1^) shows that they separately correspond to 2.0 for WT, 1.0 for S322A, and 0.25 for the rest of point‐mutants (Figure 5F).

Finally, we applied MALDI‐TOF mass spectrometry to further detect structural alteration of lipid A species from the *E. coli* MG1655 expressing *nmcr‐1* (Figure S6, Supporting Information). Unlike the fact that a single peak of lipid A (*m/z*, 1797.172–1797.307) is present in the two negative‐control strains (MG1655 alone (Figure S7A, Supporting Information) or carrying the empty vector pBAD24 (Figure S7B, Supporting Information)), an additional unique peak (*m/z*, 1920.324) of PEA‐4′‐lipid A, the modified form of lipid A, appears the *nmcr‐1*‐harboring *E. coli* (Figure S7C, Supporting Information). As expected, none of all the five mutants (namely, E239A (Figure S7G, Supporting Information), T277A (Figure S7H, Supporting Information), H377A (Figure S7L, Supporting Information), D452A (Figure S7N, Supporting Information), and H453A (Figure S7O, Supporting Information)) of NMCR‐1 with an inactivation in catalytic sites (Figure 5A) can modify the lipid A moieties of LPS anchored on bacterial surface because the only one peak (*m/z*, 1796.459–1796.668), referring to an intact lipid A, consistently occurs in the aforementioned strains. Whereas for the eight PE cavity‐forming residues (Figure 5B), the alanine substitution of S322A (Figure S7J, Supporting Information) is the only one that retains partial activity in the transfer of PEA (*m/z*, 123) to the suggestive phosphate position of lipid A (*m/z*, 1796.633), giving PEA‐4′‐lipid A (*m/z*, 1920.000). In contrast, all the remaining seven point‐mutants of NMCR‐1 are enzymatically inactive, which include N103A, T107A & E111A (Figure S7D–F, Supporting Information), N320A (Figure S7I, Supporting Information), K325A (Figure S7K, Supporting Information), H382A (Figure S7M, Supporting Information), and H465A (Figure S7P, Supporting Information), respectively. Undoubtedly, these results represent a functional proof that the PE cavity does play an essential role in the NMCR‐1‐mediated resistance to bacterial killing by colistin.

### 2.5 Interdomain Crosstalk among NMCR‐1, MCR‐1, and MCR‐4

The MCR family of enzymes comprises a TM domain that is connected with a catalytic domain by a flexible linker (Figure 1A, and Figures S1A and S6A, Supporting Information). A recent study by Sun and co‐workers^12^ elucidated that these two motifs of MCR‐1 are functionally exchangeable with the counterparts in MCR‐2. This verified the phylogenetic position of being placed into the same lineage of Subclade I (Figure 3B). In contrast, TM and catalytic domain of MCR‐1/2 cannot be equivalent to that of MCR‐3.^27^ It is consistent with the location of MCR‐3 in Subclade II distinct from Subclade I occupied by MCR‐1/2 (Figure 3B). Given that both NMCR‐1 of *Shewanella* and MCR‐4 are located within the same Subclade II, we therefore anticipated that interdomain communication occurs between NMCR‐1 and MCR‐4 (but not MCR‐1).

To examine this speculation, we engineered four hybrid (domain‐swapped) versions of NMCR‐1 using the method of overlapping PCR as recently described.^11^, ^45^ As a result, our chimeric versions separately refer to TMN1‐MCR‐1 (a derivative of MCR‐1 whose TM region is replaced with the counterpart of NMCR‐1), TM1‐NMCR‐1 (a derivative of NMCR‐1 whose TM region is replaced with the counterpart of MCR‐1), TMN1‐MCR‐4 (a derivative of MCR‐4 whose TM region is replaced with the counterpart of NMCR‐1), and TM4‐NMCR‐1 (a derivative of NMCR‐1 whose TM region is replaced with the counterpart of MCR‐4) (**Figure** **6**A). Western blot assay confirmed that as the original version, all the aforementioned 4 hybrid *mcr*‐like genes are expressed in *E. coli* (Figure 6B). In antibiotic resistance assays, the two constructs (TMN1‐MCR‐1 and TM1‐NMCR‐1) are inactive (Figure 6C), indicating that the TM domain of NMCR‐1 is incompatible with the catalytic domain of MCR‐1 and vice versa. However, the expression of TMN1‐MCR‐4 and TM4‐NMCR‐1 in *E*. *coli* can allow the recipient strain of *E. coli* MG1655 resistant to grow on the LBA plates supplemented with colistin at the level of up to 2 and 8 µg mL^−1^ (Figure 6C). Evidently, the TM1 domain of NMCR1 is compatible with the catalytic domain of MCR‐4 and vice versa (Figure 6A,C). This was further confirmed by structural determination of LPS‐lipid A pools from *E. coli* carrying derivatives of *nmcr‐1* (Figure 6D–L). MALDI‐TOF mass spectrometry showed that i) both TMN1‐MCR‐1 (Figure 6I) and TM1‐NMCR‐1 (Figure 6J) lose the enzymatic activity of modifying lipid A with an addition of PEA, similar to scenarios seen with the two negative‐control strains, MG1655 alone (Figure 6D) or bearing the empty vector (Figure 6E); ii) both TMN1‐MCR‐4 (Figure 6K) and TM4‐NMCR‐1 (Figure 6L) remain functional in catalyzing the transfer of PEA to the suggestive 4′‐phosphate position of lipid A moieties, which is almost identical to those of the parental version, MCR‐1 (Figure 6F), MCR‐4 (Figure 6G), and NMCR‐1 (Figure 6H). In summary, the distinction of interdomain interactions among NMCR‐1, MCR‐4, and MCR‐1 further suggested the diversity of evolutionary paths across the entire family of MCR‐like enzymes.

### 2.6 Impact of NMCR‐1 on Metabolic Fitness in *E. coli*

**Figure 7:**
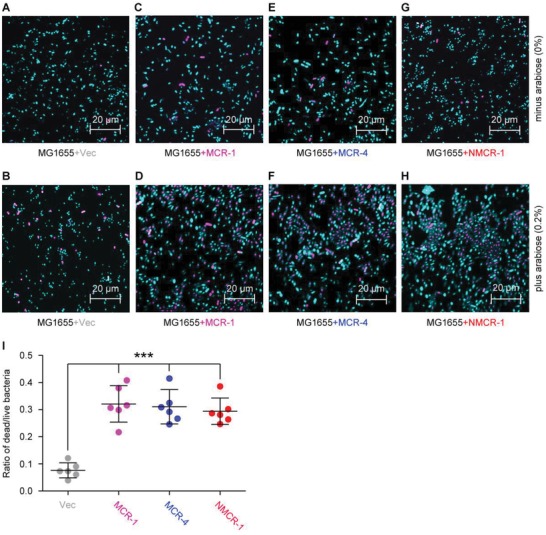
Fitness cost caused by NMCR‐1 in *E. coli*. Use of confocal microscopy to visualize the bacterial viability of the negative‐control strain (*E. coli* MG1655 with the empty vector) maintained on the growth condition A) without arabinose or B) with the addition of 0.2% w/v L‐arabinose. Similar to scenarios of the two distinct mobile colistin resistance members C) *mcr‐1* and E) *mcr‐4*, the nonmobile agent *nmcr‐1* cannot influence bacterial viability of G) *E. coli*, if it appears on the media without the induction of L‐arabinose. The addition of 0.2% w/v L‐arabinose into LB media augments functional expression of D) *mcr‐1*, F) *mcr‐4*, and H) *nmcr‐1*, giving inhibitory effects on bacterial viability. A representative result from three independent experiments is given. I) Measurement of the relative ratio of LIVE/DEAD *E. coli* strains with and/or without expression of either NMCR‐1 or MCR‐1/4. To figure out LIVE/DEAD cells, LIVE/DEAD kit is applied in bacterial staining, and confocal laser scanning microscopy is adopted to produce the images in which light blue and magenta refer to live and dead cells. The data were expressed using one‐way analysis of variance (ANOVA) followed by a Tukey–Kramer multiple comparison post hoc test.^77^ Statistical significance was set at ***p < 0.001.

To wonder whether or not the expression of *nmcr‐1* (an intrinsic agent of colistin resistance) poses an impact on bacterial metabolism, we engineered a recombinant strain of *E. coli* (FYJ1491), harboring an arabinose‐inducible plasmid‐borne *nmcr‐1*, pBAD24‐8xHis*::nmcr‐1* (Table S1, Supporting Information). First, it was assayed in growth curves (Figure S8, Supporting Information). Regardless of the vector pBAD24‐8xHis, bacterial growth of MG1655 strain is not significantly influenced by the supplementation of arabinose at the level of up to 0.2 mg mL^−1^ (Figure S8A,B, Supporting Information). In contrast, the expression of *mcr‐1* (Figure S8C, Supporting Information) and *mcr‐4* (Figure S8D, Supporting Information) is activated upon the concentration of arabinose is increased to 0.2%, which in turn inhibits bacterial growth. As predicted, a similar scenario was also seen with NMCR‐1 (Figure S8E, Supporting Information), because 0.2% arabinose‐driven production of *nmcr‐1* exerted comparable level of toxic effect on the growth of *E. coli* MG1655 (Figure S8E, Supporting Information). Second, we applied confocal microscopy to examine bacterial viability of the aforementioned strains following being assays in LIVE/DEAD staining (**Figure** **7**). No obvious bacterial death was seen as for the following two combinations, namely, i) a given *E. coli* strain on LBA medium without any addition of arabinose (Figure 7A,C,E,G) and ii) empty vector‐containing MG1655 regardless of arabinose (Figure 7A,B). In contrast, dead cells were clearly visible in *nmcr‐1*‐expressing *E. coli* (Figure 7H,I), at a comparable level to those of MCR‐1 (Figure 7C,D) and MCR‐4 (Figure 7E,F). Therefore, we concluded that *E. coli* exhibits a significant fitness cost to respond the stress by NMCR‐1, a nonmobile colistin resistance determinant, as a transferable member of MCR family does.

## 3 Discussion

As a defense weapon, the majority of bacterial colistin resistance arises from charge‐related structural changes of LPS‐lipid A in *Enterobacteriaceae*. To our surprise, not only does the CptC of *Campylobacter* (a chromosomally encoded EptA homolog) decorate the LPS‐lipid A, but also modifies its flagella rod subunit protein FlgG.^64^ The bifunctional role of EptC contributed greatly to bacterial virulence and anti‐host innate immunity in phagocytosis.^65^, ^66^, ^67^ It is of much interest to test whether or not the NMCR‐1 family of determinants plays similar roles in protein post‐translational modification and bacterial pathogenesis. The genus of *Shewanella* refers to a group of gram‐negative bacteria inhabiting in marine environment or aquatic ecosystem.^68^, ^69^ Among the 67 different known species of this genus, *S. oneidensis* MR‐1 is a model organism with remarkably metabolic versatility, possessing robust potential for bioremediation of the heavy metals‐contaminated environment.^69^ By contrast, certain species of *Shewanellaceae*, such as *S. algae* 55, 68, 70 and *S. putrefaciens*,^70^, ^71^ are recognized as opportunistic pathogens, causing the skin and soft‐tissue infections in humans with occupational exposure or recreational activities to marine niches.^68^ More importantly, increasingly accumulated evidence has indicated that several species of *Shewanella* from environmental and clinical settings harbor progenitor determinants for certain antimicrobial resistance and thereafter display extensive phenotypic multidrug resistance.^72^, ^73^ A *qnrA*‐like gene, encoding quinolone resistance, has been detected in *S. algae* 74 as well as the clinical isolate *S*. sp. JAB‐1^75^ and *Shewanella xiamenensis*.^76^ Obviously, it poses the possibility of aquaculture as a reservoir/source for certain antibiotic resistance.

The data we showed here define NMCR‐1, a new family of nonmobile colistin resistance determinants (Figure 3). Similar to ICR‐Mo, a progenitor of MCR‐1/2 colistin resistance,^49^ NMCR‐1 also interferes production of de novo reactive oxygen species stimulated by colistin. Because growth retardation is an indicative of bacterial death as a fitness cost, it was observed initially with MCR‐1,^77^ MCR‐2,^27^ MCR‐3,^27^ and MCR‐4.^46^ Thus, it is not unexpected that the reduction of bacterial survival proceeds to balance *nmcr‐1* expression in *E. coli* (Figure S8G–I, Supporting Information) at the level equivalent to those of MCR‐1 (Figure S8A–D, Supporting Information) and MCR‐4 (Figure S8E,F, Supporting Information). The compatibility of the TM and catalytic domains exclusively in NMCR‐1 versus MCR‐4, but not NMCR‐1 versus MCR‐1 (Figure 6), allowed us to more confidently conclude that NMCR‐1 is a progenitor of MCR‐4. Taken together, this finding supplements an additional example that the aquatic bacterium *Shewanella* (and even its associated aquaculture^78^) is a major source for diversified determinants of antibiotic resistance. In general, this is also consistent with our recent proposal that functional gain of MCR‐4 proceeds via progressive evolution of an inactive MCR‐4.3.^46^ Along with the recognition of MCR‐1/2 progenitor,^49^ our mechanistic understanding of the MCR‐4 origin paves a way for future efforts to screen and/or design compounds of small molecule that has the robust potential of reversing colistin resistance. Thereafter, it might be informative for us to reconsider the possible origin of MCR‐4 in the context of one health approach with destination to efficient intervention and even control of global dissemination of mobile colistin resistance.

## 4 Experimental Section

*Genetic Manipulations*: The full‐length *nmcr‐1* gene was amplified from *S*. *algae* MARS14 (Table S1, Supporting Information), and cloned into an arabinose‐inducible expression vector pBAD24‐8xHis, an improved version of pBAD24. Also, an alternative version of *nmcr‐1* was synthesized with optimized codons. Both of them were tested in the context of colistin resistance. As recently described,^11^, ^12^ overlapping PCR experiments were conducted to give mosaic genes of *nmcr‐1* (Table S1, Supporting Information). This allowed to further domain‐swapping assays for *nmcr‐1*, *mcr‐1*, and *mcr‐4*. The point‐mutants of *nmcr‐1* were produced using the Mut Express II fast mutagenesis kit version 2 (Vazyme Biotech Co., Ltd.) with appropriate primers (Table S1, Supporting Information). Of note, the recombinant plasmid pHGE‐Ptac*::nmcr‐1* was transferred into the *E. coli* donor strain WM3064 (a DAP auxotroph), and then mated into the recipient *S. oneidensis* via a routine conjugation. All the plasmid constructs were verified by direct DNA sequencing.

*Determination of Bacterial Viability and Colistin Resistance*: Bacterial viabilities of *E. coli* MG1655 carrying *nmcr‐1* and its derivatives were determined using the method of solid LBA broth dilution test.^22^, ^23^, ^79^ Briefly, the mid‐log phase cultures were diluted in series of tenfold, spotted on LBA plates containing varied level of colistin (0.0, 0.5, 1.0, 2.0, 4.0, 8.0, 16.0 to 32.0 µg mL^−1^), and maintained at 37 °C for over 20 h. To measure the MIC of colistin in *E. coli*, liquid broth dilution tests were conducted as recommended by European Committee on Antimicrobial Susceptibility Testing (EUCAST) with Cation‐Adjusted Mueller‐Hinton Broth (CAMHB).^12^ In general, bacterial cultures were diluted 100‐fold in CAMHB containing varied level of colistin and kept in the shaker at 200 rpm at 37 °C for 16 h. As recently conducted with the biotin regulator BioQ of *Mycobacterium*,^80^ functional expression of *nmcr‐1* and derivatives in the aforementioned strains (Table S1, Supporting Information) was routinely verified using western blot with anti‐6x his primary antibody.

*Measurement of Metabolic Fitness Cost*: To elucidate effects of *nmcr‐1* (and/or *mcr‐1*/*4*) on bacterial metabolism, bacterial growth curves of the *E. coli* MG1655 alone (or carrying the pBAD24‐borne *nmcr‐1* (and/or *mcr*‐like genes)) were measured at 37 °C (180 rpm). The optical density at the wavelength of 600 nm (OD600) was recorded with spectrophotometer (Spectrum lab S32A) at a regular interval of 1 h for a total period of 20 h.^27^

In addition, the *E. coli* cultures were stained in LIVE/DEAD assays and then subjected to the confocal microscopy‐aided visualization of bacterial viability as recently reported.^27^, ^77^ The images were taken by the confocal laser scanning microscopy (Zeiss LSM 800) with a 63 × oil immersion lens and analyzed using COMSTAT image analysis software. The Tukey–Kramer multiple comparison post hoc test was performed to value the COMSTAT data. Statistical significance was set at *p* < 0.01 with *t*‐test.

*Preparation and Identification of NMCR‐1 Membrane Protein*: The *E. coli* strain of BL21(DE3) pLysS carrying pBAD24‐8xHis*::nmcr‐1* was used for protein production in which 0.2% (vol/vol) arabinose acted as an inducer. Bacterial pellet was resuspended with buffer A (20 × 10^−3^ m Tris‐HCl (pH 8.0), 100 × 10^−3^ m NaCl, 5 × 10^−3^ m DNaseI, 1 × 10^−3^ m phenylmethylsulfonyl‐fluoride), 2 × 10^−3^ m MgCl_2_, and 10% glycerol) at 4 °C. After three rounds of lysis by French pressure, bacterial debris was removed by centrifugation (16 800 rpm for 1 h), and the resultant supernatant was subjected to the second round of centrifugation (38 000 rpm, 1.5 h). The pellet containing NMCR‐1 membrane protein was solubilized in buffer B (20 × 10^−3^ m Tris‐HCl (pH 8.0), 100 × 10^−3^ m NaCl, 10% glycerol, and 1% DDM), and then centrifuged (38 000 rpm for 1.5 h) for further clarification. Following 4 h of incubation of the soluble fraction with the nickel‐nitrilotriacetic acid agarose (Qiagen) pre‐equilibrated with wash buffer (20 × 10^−3^ m Tris‐HCl (pH 8.0), 100 × 10^−3^ m NaCl, 10% glycerol, 0.03% dodecyl‐β‐*D*‐maltoside (DDM), 20 × 10^−3^ m imidazole), the NMCR‐1 protein of interest was washed with wash buffer containing 50 × 10^−3^ m imidazole, and then eluted with elution buffer (20 × 10^−3^ m Tris‐HCl (pH 8.0), 100 × 10^−3^ m NaCl, 10% glycerol, and 0.03% DDM) carrying 100 × 10^−3^ m imidazole as recently described.^11^, ^23^, ^27^, ^45^, ^49^ Finally, the purified NMCR‐1 protein was concentrated with a 30 kDa cut‐off ultrafilter column (Millipore), and judged with sodium dodecyl sulfate‐polyacrylamide gel electrophoresis (SDS‐PAGE) (12%) staining with Coomassie Brilliant Blue R‐250 dye.

The identity of protein with expected size was determined with A Waters Q‐Tof API‐US Quad‐ToF mass spectrometry.^81^, ^82^ The secondary structure of NMCR‐1 was illustrated with an approach of CD. The NMCR‐1 protein was dissolved in the buffer (20 × 10^−3^ m Tris‐HCl (pH 8.0), 100 × 10^−3^ m NaCl, 5% glycerol, and 0.03% DDM) and subjected to subsequent analysis of folding mode with a Jasco Model J‐1500 spectrometer (Jasco Corp., Tokyo, Japan). The data were recorded through continuous scanning of wavelength from 190 to 240 nm.^83^, ^84^ To further probe whether or not zinc ions are occupied with NMCR‐1 protein, ICP‐MS was applied as recently described.^27^, ^49^ Along with the carrier gas of helium, NMCR‐1 protein (≈0.2 mg mL^−1^) was loaded into an NexION 300X ICP‐MS instrument (PerkinElmer, USA) for the determination of ICP‐MS.^49^, ^85^

*Enzymatic Reaction of NMCR‐1 In Vitro*: An in vitro system of NMCR‐1 reaction was established as Anandan et al.^42^ initially reported with little change. The reaction catalyzed by NMCR‐1 was maintained at room temperature for 24 h. In this system (50 µL in total, 50 × 10^−3^ m HEPES (pH 7.5), 100 × 10^−3^ m NaCl, 0.03% of DDM), NBD‐glycerol‐3‐PEA is an alternative substrate for the NMCR‐1 enzyme. Of note, it is abbreviated from 1‐acyl‐2‐{12‐[(7‐nitro‐2‐1,3‐benzoxadiazol‐4‐yl) amino] dodecanoyl},‐sn‐glycero‐3‐phosphoethanolamine (Avanti Lipids, USA).

As described with MCR‐1/2^11^, ^45^ and ICR‐Mo,^49^ TLC was carried out to distinguish the product of NBD‐glycerol from the reactant of NBD‐glycerol‐3‐PEA. To furthermore validate the transfer of PEA catalyzed by NMCR‐1, the reaction mixture was subjected to the chemical identification with liquid chromatography mass spectrometry (Agilent technologies 6460 Triple Quad LC/MS) for chemical identity.^86^, ^87^

*Sequence Data*: GenBank accession numbers of the new gene NMCR‐1 and its six variants that were renamed in this study are listed as follows: MH909845 (the former Acc. no.: LN811438) for NMCR‐1 of *S. algae*; MH105287 (the former Acc. no.: WP028779212) for NMCR‐1.2 of *Shewanella* sp. 38A_GOM‐205m; MH105288 (the former Acc. no.: WP 071237666) for NMCR‐1.3 of *S. algae*; MH105289 (the former Acc. no.: WP101058799) for NMCR‐1.4 of *S. upenei*; MH105290 (the former Acc. no.: WP101056665) for NMCR‐1.5 of *S. chilikensis*; MH105291 (the former Acc. no.: WP101096436) for NMCR‐1.6 of *S*. *indica*; MH105292 (the former Acc. no.: WP039033020) for NMCR‐1.7 of *S*. sp. ECSMB14102; and MH822841 (the former Acc. no.: NZ_NGVS01000023) for NMCR‐1.8 of *S. carassii* strain 08MAS2251, respectively.

## Conflict of Interest

The authors declare no conflict of interest.

## Supporting information

Supplementary
