# Correction to “Definition of a Family of Nonmobile Colistin Resistance (NMCR‐1) Determinants Suggests Aquatic Reservoirs for MCR‐4”

**DOI:** 10.1002/advs.202520184

**Published:** 2025-12-12

Zhang H, Wei W, Huang M, Umar Z, Feng Y. Definition of a Family of Nonmobile Colistin Resistance (NMCR‐1) Determinants Suggests Aquatic Reservoirs for MCR‐4. *Adv*. *Sci*. 2019, *6* (11), 1900038 (https://doi.org/10.1002/advs.201900038).

On Sep. 9, 2020, we replaced Figure 7 (https://doi.org/10.1002/advs.202002530), and this is because we found an error in the preparation, i.e., overlap of different fields of confocal microscopy. Although Figure 7F,H have been corrected, I recently noticed Figure 7D is an omission, which should be replaced. Here, we provided an update of Figure 7, as well as raw data when necessary.

Although this unintentional mistake does not affect the conclusion of this paper, we do apologize for the resultant confusion.

We apologize for this error.


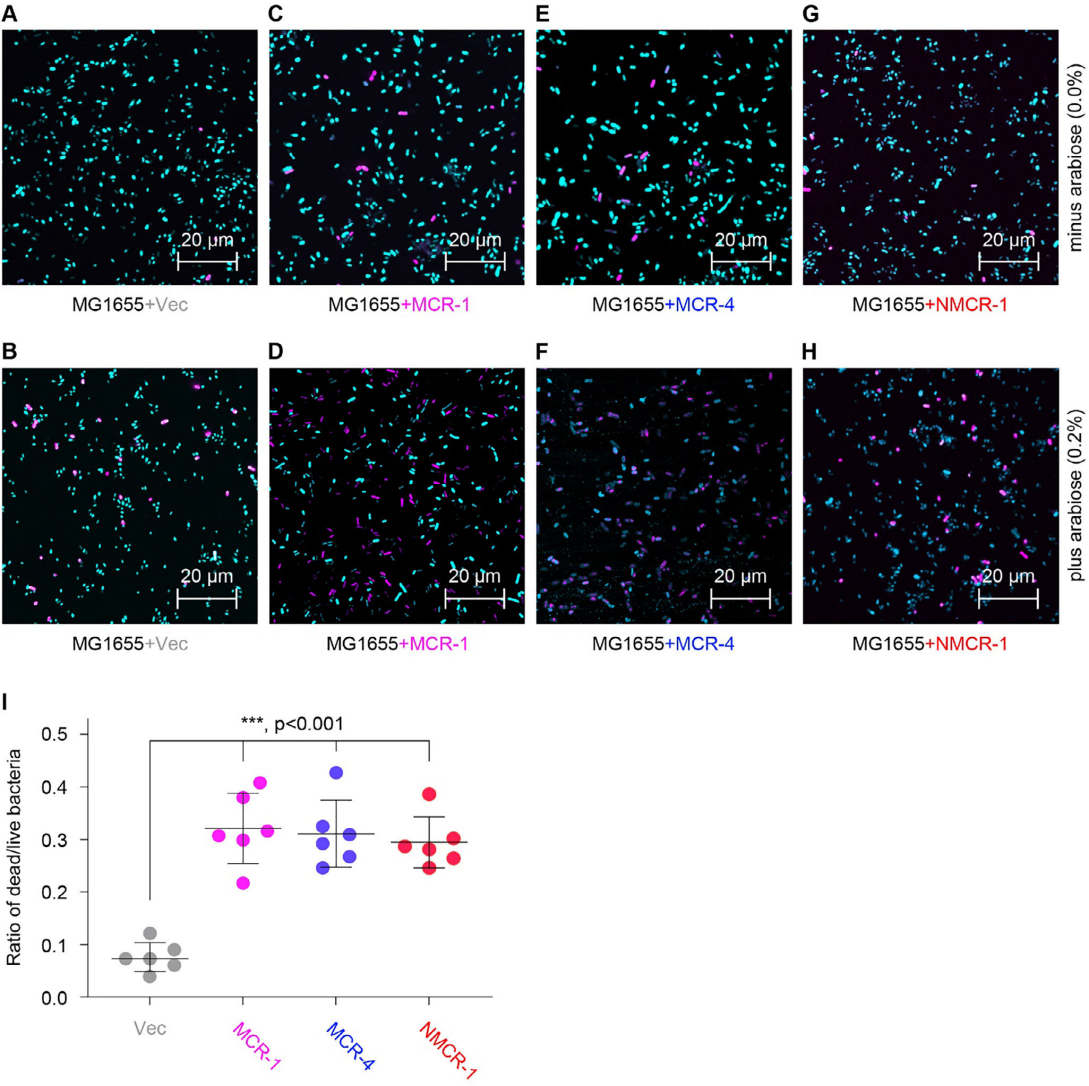


**Figure 7_rev**: Fitness cost caused by NMCR‐1 in *E. coli*

