# Supplementary material for: Definition of a Family of Nonmobile Colistin Resistance (NMCR‐1) Determinants Suggests Aquatic Reservoirs for MCR‐4
Source: Adv Sci (Weinh). 2019 Apr 3;6(11):1900038. doi: 10.1002/advs.201900038 (PMC6548957; doi:10.1002/advs.201900038)
Supplement: Supplementary file 1 — Supplementary [file ADVS-6-1900038-s001.pdf]

Copyright WILEY-VCH Verlag GmbH & Co. KGaA, 69469 Weinheim, Germany, 2019.

ADVANCED SCIENCE
Open Access

Supporting Information

for *Adv. Sci.,* DOI: 10.1002/advs.201900038

Definition of a Family of Nonmobile Colistin Resistance (NMCR-1) Determinants Suggests Aquatic Reservoirs for MCR-4

*Huimin Zhang, Wenhui Wei, Man Huang, Zeeshan Umar, and Youjun Feng**

Supplementary Materials for

Definition of A Family of Non-Mobile Colistin Resistance (NMCR-1) Determinants Suggests Aquatic Reservoirs for MCR-4

Supplementary methods

Strains, Plasmids and Growth Conditions

In addition to the two species of *Shewanella* (*S. algae* and *S. oneidensis*), the rest of bacterial strains (**Table S1**) referred to the derivatives of *E. coli* MG1655. They were grown in either liquid Luria-Bertani (LB) broth or solid LB agar (LBA) plates at 37°C (or 30°C). When necessary, antibiotics was supplemented as follows: 100 μg/ml for ampicillin; 50 μg/ml for kanamycin; 0.3 mM for 2, 6-diaminopimelic acid (DAP); and varied level (0~32μg/ml) of colistin. The strain of *E. coli* DH5α was used for gene cloning, and the strain of BL21(DE3)/pLysS acted as a host for protein expression. pBAD24-8×His refers to a derivative of pBAD24 we developed, in which a C-terminal 8x histidine tag was introduced into the SalI cut by a pair of specific primers (**Table S1**). Arabinose (0.2%) was added to activate the expression of pBAD24-borne *nmcr-1* in *E. coli*, and 0.2 mM isopropyl-β-D-thiogalactopyranoside (IPTG) was supplemented to promote the pHGE-Ptac vector-based genetic complementation of *nmcr-1* in the *S. oneidensis* (*1*).

Extraction, Purification and Identification of LPS-lipid A

Similar to the description by Liu and coworkers (*2*), lipopolysaccharide (LPS)-anchored lipid A (LPS-lipid A) was extracted from the derivatives of *E. coli* MG1655 with or whithout *nmcr-1* (or *mcr*-like genes). The LPS samples validated with silver staining were subjected to structural determiantion by MALDI-TOF mass spectrometry (Bruker, ultrafleXtreme) in negative ion mode with the linear detector. Finally, MS spectrum was given from an average of 500 shots and 50% laser power.

Structure Modeling and Molecular Docking

The modelled structure of full-length NMCR-1 was generated with the software Swiss-Model (https://swissmodel.expasy.org/interactive/qMEvX5/models/) (*3*), in which the template referred to EptA of *Neisseria meningitidis* [PDB: 5FGN] (*4*). The score of coverage combined with the value of QMEAN (that provides a global and local absolute quality estimate on the modeled structure (*5*)) are integrated to judge the qualification of this structural prediction.

UCSF DOCK 6.7 software (version 6.7) (*6*) was adopted to conduct molecular docking of NMCR-1 enzyme to its PE lipid substrate. The ready-to-dock 3D structure of phosphatidylethanolamine (PE) (ID: ZINC32837871) and its head group (ID: ZINC02798545) was deposited into ZINC database (*7*). Concrete optimization of NMCR-1 structure proceeded by using UCSF Chimera software (*8*). Given that NMCR-1 appears in the stringent state in this prediction, and can't offer enough cavity space to hold the full PE molecule with six flexible acyl chains, we thus applied the single head group of PE molecule into docking into NMCR-1 structure. The diagram for two-dimension ligand-protein interaction was produced with the LigPlot+ software (*9*).

Phylogenetic Analyses

The MCR family of colistin resistance enzymes were downloaded from GenBank database, which covers eight different subtypes (from MCR-1 to MCR-8). Each subtype involves an array of heterogeneous variants. Using Clustal Omega (http://www.ebi.ac.uk/Tools/msa/clustalo/), multiple sequence alignment of the MCR members was performed (*10*), giving its output of phylogeny with TreeView (*11*). In addition to several variants of MCR-1/2, a couple of close relatives of MCR-1 (designated as MCR-M) are also detected in certain species of *Moraxella*. The sulfatase of *Paenibacillus* sp. UNC451MF (Acc. no.: WP_028552638) was anticipated to be an origin of the MCR phylogeny.

Supplementary tables

Table S1 Bacteria, plasmids and primers used in this study

Strains/plasmids	Relevant characteristics	Origins
**Strains**		
DH5α	A cloning host of *E. coli*	Lab stock
MG1655	A wild-type strain of *E. coli*	Lab stock
FYJ795	MG1655 carrying pBAD24*::mcr-1*	Lab stock (*12*, *13*)
FYJ915	BL21 carrying pET21a*::mcr-1*	Lab stock (*12*, *13*)
FYJ1513	MG1655 carrying pBAD24-8×His	Lab stock
FYJ1413	MG1655 carrying pBAD24-8×His*::mcr-4*	Lab stock
FYJ1491	MG1655 carrying pBAD24-8×His*::nmcr-1*	This work
FYJ1492	BL21(pLysS) carrying pBAD24-8×His*::nmcr-1*	This work
FYJ514	*Shewanella algae*	This work
FYJ1493	*S. oneidensis*	Lab stock (*14*, *15*)
FYJ1494	*S. oneidensis* carrying pHGE-Ptac*::nmcr-1*	This work
FYJ1495	A donor strain of *E. coli* WM0364 for conjugation, Δ*dapA*	Gao's lab (*1*)
FYJ1496	MG1655 carrying pBAD24-8×His*::nmcr-1*(N103A)	This work
FYJ1497	MG1655 carrying pBAD24-8×His*::nmcr-1*(T107A)	This work
FYJ1498	MG1655 carrying pBAD24-8×His*::nmcr-1*(E111A)	This work
FYJ1499	MG1655 carrying pBAD24-8×His*::nmcr-1*(E239A)	This work
FYJ1500	MG1655 carrying pBAD24-8×His*::nmcr-1*(T277A)	This work
FYJ1501	MG1655 carrying pBAD24-8×His*::nmcr-1*(N320A)	This work
FYJ1502	MG1655 carrying pBAD24-8×His*::nmcr-1*(S322A)	This work
FYJ1503	MG1655 carrying pBAD24-8×His*::nmcr-1*(K325A)	This work
FYJ1504	MG1655 carrying pBAD24-8×His*::nmcr-1*(H377A)	This work
FYJ1505	MG1655 carrying pBAD24-8×His*::nmcr-1*(H382A)	This work
FYJ1506	MG1655 carrying pBAD24-8×His*::nmcr-1*(D452A)	This work
FYJ1507	MG1655 carrying pBAD24-8×His*::nmcr-1*(H453A)	This work

FYJ1508	MG1655 carrying pBAD24-8×His::*nmcr-1*(H465A)	This work
FYJ1509	MG1655 carrying pBAD24-8×His::tmn1-*mcr-1*	This work
FYJ1510	MG1655 carrying pBAD24-8×His::tm1-*nmcr-1*	This work
FYJ1511	MG1655 carrying pBAD24-8×His::tmn1-*mcr-4*	This work
FYJ1512	MG1655 carrying pBAD24-8×His::tm4-*nmcr-1*	This work
**Plasmids**		
pBAD24	An arabinose inducible expression vector with C-terminal 8×His; Amp^R	Lab stock
pHGE-Ptac	An IPTG-inducible P_{tac} expression vector, Km^R	Gao's lab (*1*)
pHGE-Ptac::*nmcr-1*	A recombinant pHGE-Ptac carrying the wild-type *nmcr-1* at the two cuts of EcoRI and SacI; Amp^R	This work
pBAD24::*mcr-1*	A pBAD24-8×His carrying *mcr-1* at the two cuts of EcoRI and SalI; Amp^R	Lab stock (*12, 13*)
pBAD24::*mcr-4*	A pBAD24-8×His carrying *mcr-4* at the two cuts of EcoRI and SalI; Amp^R	Lab stock
pBAD24::*nmcr-1*	A pBAD24-8×His carrying *nmcr-1* at the two cuts of EcoRI and SalI; Amp^R	This work
pBAD24::*nmcr-1* (N103A)	pBAD24-8×His encoding the mutant version of *nmcr-1*(N103A); Amp^R	This work
pBAD24::*nmcr-1* (T107A)	pBAD24-8×His encoding the mutant version of *nmcr-1*(T107A); Amp^R	This work
pBAD24::*nmcr-1* (E111A)	pBAD24-8×His encoding the mutant version of *nmcr-1*(E111A); Amp^R	This work
pBAD24::*nmcr-1* (E239A)	pBAD24-8×His encoding the mutant version of *nmcr-1*(E239A); Amp^R	This work
pBAD24::*nmcr-1* (T277A)	pBAD24-8×His encoding the mutant version of *nmcr-1*(T277A); Amp^R	This work
pBAD24::*nmcr-1* (N320A)	pBAD24-8×His encoding the mutant version of *nmcr-1*(N320A); Amp^R	This work
pBAD24::*nmcr-1* (S322A)	pBAD24-8×His encoding the mutant version of *nmcr-1*(S322A); Amp^R	This work
pBAD24::*nmcr-1* (K325A)	pBAD24-8×His encoding the mutant version of *nmcr-1*(K325A); Amp^R	This work
pBAD24::*nmcr-1* (H377A)	pBAD24-8×His encoding the mutant version of *nmcr-1*(H377A); Amp^R	This work
pBAD24::*nmcr-1* (H382A)	pBAD24-8×His encoding the mutant version of *nmcr-1*(H382A); Amp^R	This work
pBAD24::*nmcr-1* (D452A)	pBAD24-8×His encoding the mutant version of *nmcr-1*(D452A); Amp^R	This work
pBAD24::*nmcr-1* (H453A)	pBAD24-8×His encoding the mutant version of *nmcr-1*(H453A); Amp^R	This work

pBAD24::*nmcr-1* (H465A)	pBAD24-8×His encoding the mutant of *nmcr-1*(H465A); Amp^R	This work
pBAD24::tmn1-*mcr-1*	pBAD24-8×His expressing a hybrid derivative "tmn1-*mcr-1*" of *mcr-1*, whose TM region is replaced with the counterpart in *nmcr-1*; Amp^R	This work
pBAD24::tm1-*nmcr-1*	pBAD24-8×His encoding a mosaic derivative "tm1-*nmcr-1*" of *nmcr-1*, whose TM region is replaced with the counterpart in *mcr-1*; Amp^R	This work
pBAD24::tmn1-*mcr-4*	pBAD24-8×His harboring a hybrid version "tmn1-*mcr-4*" of *nmcr-4*, whose TM region is replaced with the counterpart in *nmcr-1*; Amp^R	This work
pBAD24::tm4-*nmcr-1*	pBAD24-8×His containing a hybrid version "tm4-*nmcr-1*" of *nmcr-1*, whose TM region is replaced with the counterpart in *mcr-4*; Amp^R	This work

**Primers**	**Sequences**
pBAD24-*nmcr-1*-F (EcoRI)	5'-CCG GAA TTC ATG AGC GTG CTG AAC CCG ATC-3'
pBAD24-*nmcr-1*-R (SalI)	5'-ACG CGT CGA CAC GAC GGC ACA GCG CGA TCA G-3'
nmcr-1-TM-R	5'-CAT GTA AAA GAA CGC AAT AAC CAC-3'
nmcr-1-OS-F	5'-CAG AAC TAT CTG GCG TTC GGT C-3'
pBAD24-*mcr-1*-F (EcoRI)	5'-CCG GAA TTC ATG CAG CAT ACT TCT GTG TG-3'
pBAD24-*mcr-1*-R (SalI)	5'-ACG CGT CGA CGC GGA TGA ATG CGG TGC GGT C-3'
mcr-1-TM-R	5'-CCG AAC GCC AGA TAG TTC TGA CTG GCA TAA TGA CTG CTG AAC G-3'
mcr-1-OS-F	5'-TTA TTG CGT TCT TTT ACA TGT TCT TTC GCG TGC ATA AGC CGC-3'
pBAD24-*mcr-4*-F (EcoRI)	5'-CCG GAA TTC ATG ATT TCT AGA TTT AAG ACG TTA T-3'
pBAD24-*mcr-4*-R (SalI)	5'-ACG CGT CGA CAT ACC TGC AAG GTG CAA AAA TAT-3'
pHGE-Ptac-*nmcr-1*-F (EcoRI)	5'-CCG GAA TTC ATG AGT GTG TTG AAT CCC ATC TC-3'
pHGE-Ptac-*nmcr-1*-R (SacI)	5'-CGA GCT CTC AAC GGC GAC AAA GGG CAA TC-3'
mcr-4-TM-R	5'-CCG AAC GCC AGA TAG TTC TGA TAG TAA AAA AAG GCG ACT ATC C-3'
mcr-4-OS-F	5'-TTA TTG CGT TCT TTT ACA TGC AAG ATT ATG CTG CAT TTG TTC G-3'
nmcr-1(N103A)-F	5'- TGA TCG AGG CAA CCT TCC AGA CCA ACA GCG CG-3'
nmcr-1(N103A)-R	5'- GAA GGT TGC CTC GAT CAT GCC GGT ATC AAA AA-3'

nmcr-1(T107A)-F	5’- ACC TTC CAG GCA AAC AGC GCG GAA GCG CTG AC-3’
nmcr-1(T107A)-R	5’- CTG TTT GCC TGG AAG GTG TTC TCG ATC ATG CC-3’
nmcr-1(E111A)-F	5’-AGA CCA ACA GCG CGG CAG CGC TGA CCT ACG TTA ACT GG-3’
nmcr-1(E111A)-R	5’- TGC CGC GCT GTT GGT CTG GAA GGT GTT CTC GA-3’
nmcr-1(E239A)-F	5’- TCT GGT GGT GGG TGC AAC CGC GCG TGC GCA GAA C-3’
nmcr-1(E239A)-R	5’- TTG CAC CCA CCA CCA GAA CCA TCA GAC GCG GT-3’
nmcr-1(T277A)-F	5’- TAC CGC GGC AGC TGT GAG CCT GCC GTG CAT GT-3’
nmcr-1(T277A)-R	5’- TCA CAG CTG CCG CGG TAC CGC AGC TGC TAA CA-3’
nmcr-1(S322A)-F	5’- CAA CGA TGC AGG TTG CAA GGG CGT GTG CGA CC -3’
nmcr-1(S322A)-R	5’- TGC AAC CTG CAT CGT TGT CGA ACC ATT GAA CC-3’
nmcr-1(K325A)-F	5’-ACG ATA GCG GTT GCG CAG GCG TGT GCG ACC GTG TT-3’
nmcr-1(K325A)-R	5’- TGC GCA ACC GCT ATC GTT GTC GAA CCA TTG AA-3’
nmcr-1(H377A)-F	5’- CGT GCT GGC AAT CAT TGG TAG CCA CGG CCC GA -3’
nmcr-1(H377A)-R	5’- CAA TGA TTG CCA GCA CGA TCA GAC GGT CTT GG-3’
nmcr-1(H382A)-F	5’- ATC ATT GGT AGC GCA GGC CCG ACC TAC TAT CTG CG-3’
nmcr-1(H382A)-R	5’- CCT GCG CTA CCA ATG ATG TGC AGC ACG ATC AG-3’
nmcr-1(D452A)-F	5’- TGT ATC TGA GCG CAC ACG GCG AGA GCC TGG GT-3’
nmcr-1(D452A)-R	5’- GTG TGC GCT CAG ATA CAT CAT CGC GGT ATC GT -3’
nmcr-1(H453A)-F	5’- TGT ATC TGA GCG ACG CAG GCG AGA GCC TGG GTG AA -3’
nmcr-1(H453A)-R	5’- TGC GTC GCT CAG ATA CAT CAT CGC GGT ATC GT-3’
nmcr-1(H465A)-F	5’- ATG TAC CTG GCA GGC AGC CCG TAT GCG ATT GC-3’
nmcr-1(H465A)-R	5’- CTG CCT GCC AGG TAC ATG CCC TTT TCA CCC AG-3’
nmcr-1(N320A)-F	5’- TGG TTC GAC GCA GAT AGC GGT TGC AAG GGC GT-3’
nmcr-1(N320A)-R	5’- CTA TCT GCG TCG AAC CAT TGA ACC TTC ACG CC-3’

*The underlined letters in italic denote restrictions sites

Supplementary figures

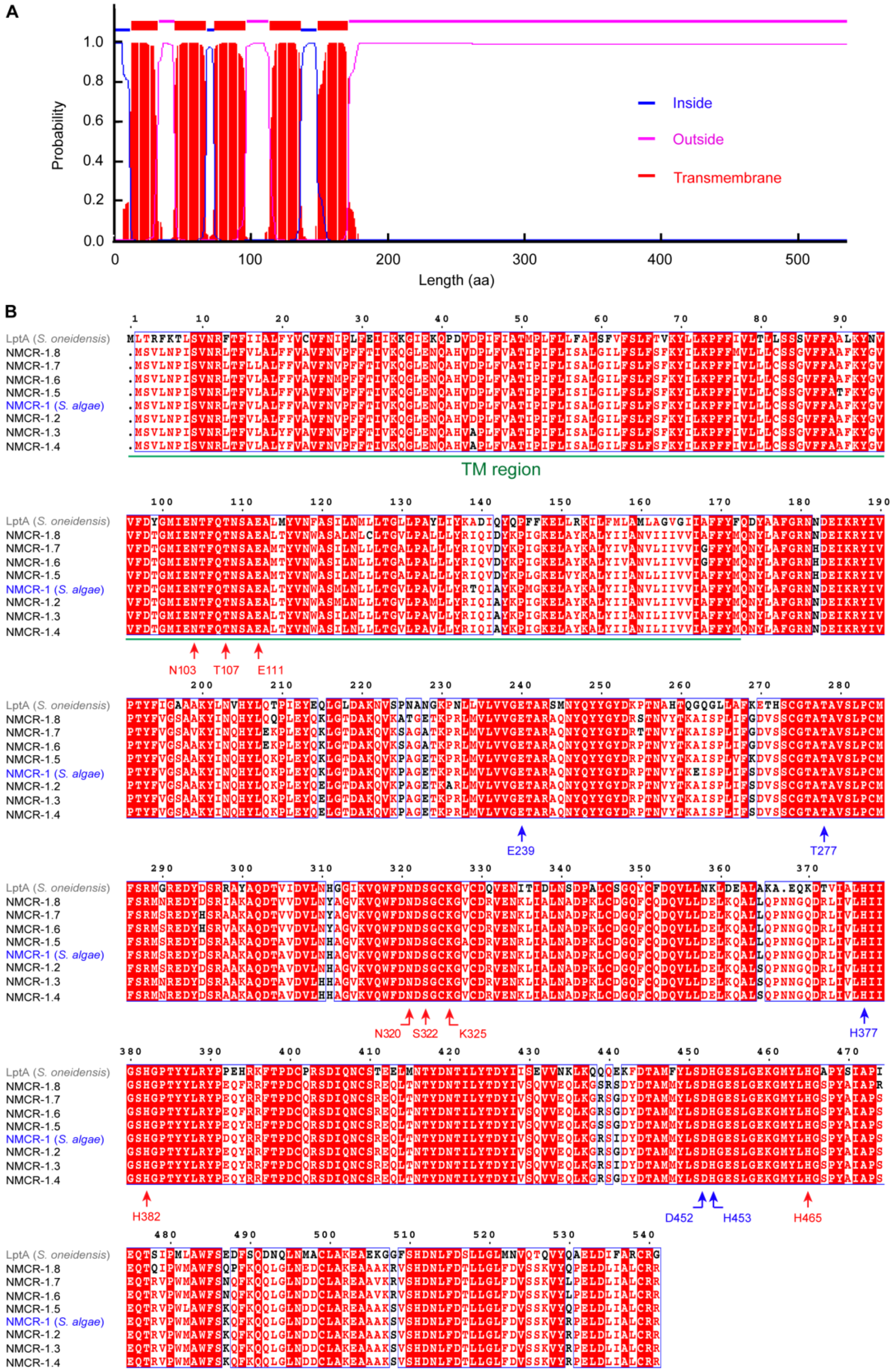

Fig. S1 Bioinformatic analyses of the new family of non-mobile colistin

resistance (NMCR-1) determinants exclusively in certain species of *Shewanella*

A. Trans-membrane prediction of NMCR-1 with the program of TMHMM

B. Multiple sequence alignment for a new family of non-mobile colistin resistance (NMCR-1) determinants with the *S. oneidensis* LptA

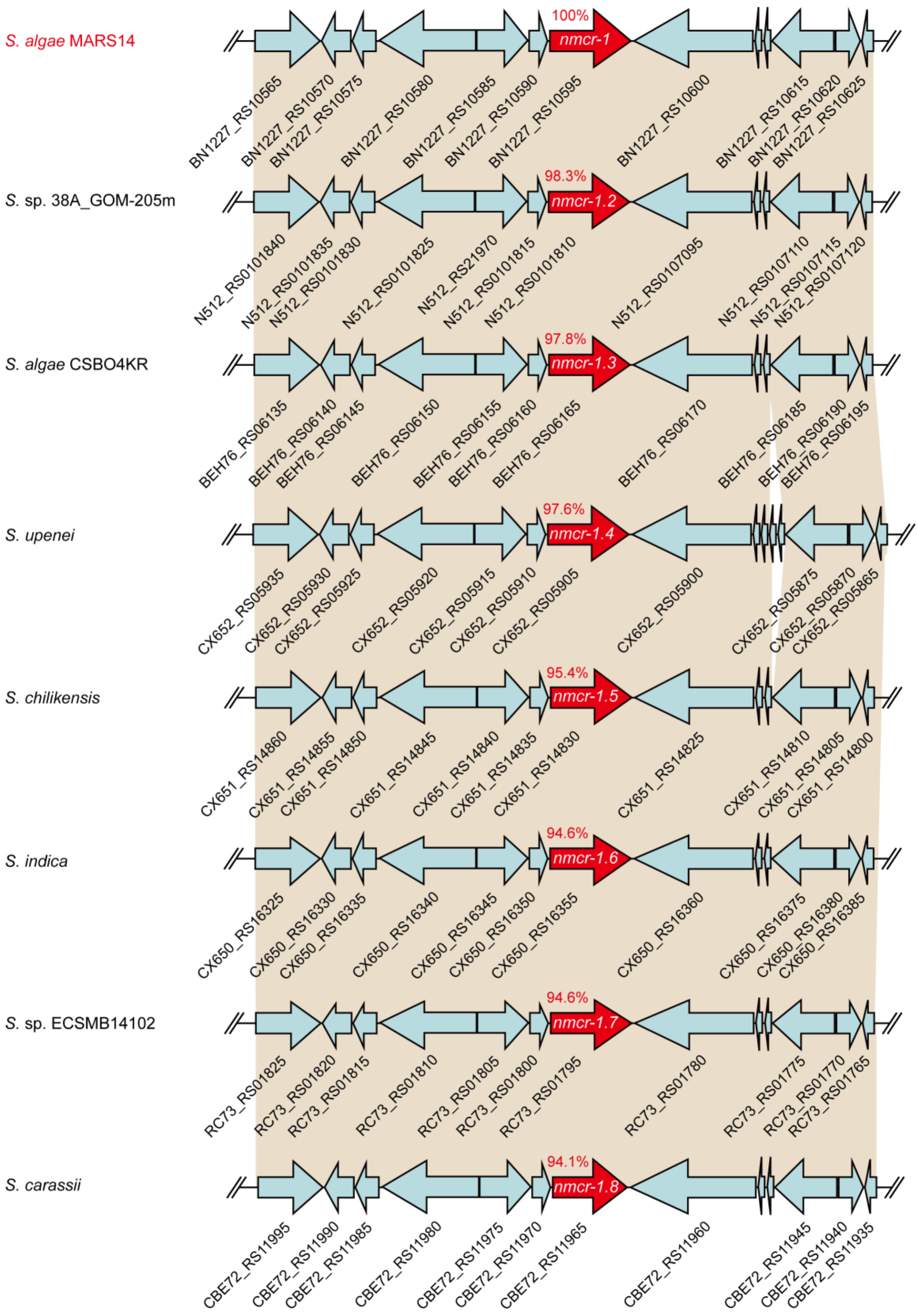

Fig. S2 Genetic context of *nmcr-1* and its variants (from *nmcr-1.2* to *nmcr-1.8*) in certain species of *Shewanella*
The nucleotide similarity of *nmcr-1* variants is labeled.

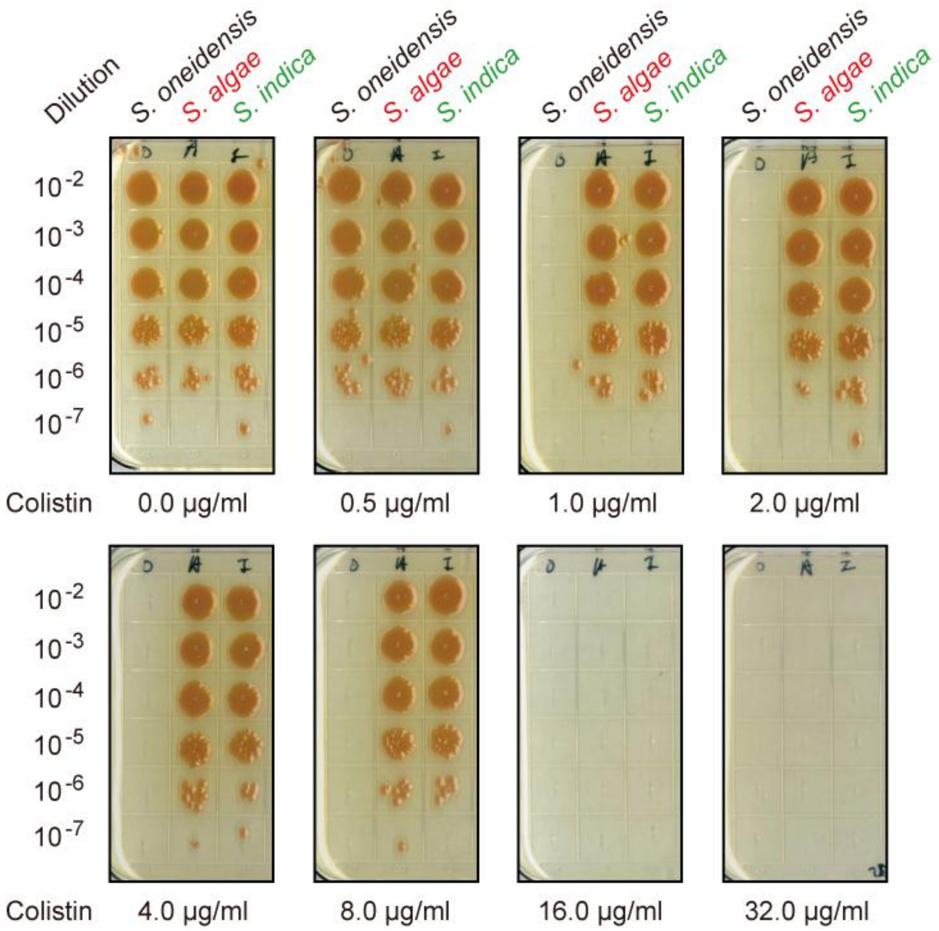

Fig. S3 *S. indica* that encodes *nmcr-1.6* displays similar level of colistin resistance to that of *S. algae* carrying *nmcr-1*

Bacterial strains were kept on LBA plates with different levels of colistin.

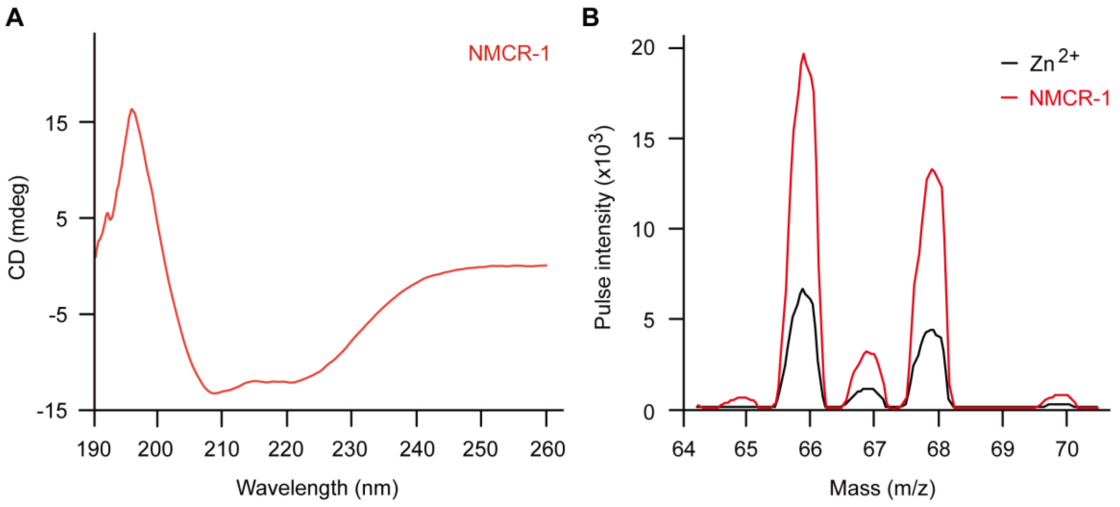

C

>NMCR-1

1 **MSVLNPISVN R**LTFVLALFF VAVFNVPFFT IVK**QGLENQA HVDPLFVATI PIFLISALGI**
61 **LFSLFSFK**YI LKPFFIVLLL CSSGVFFAAF KYGVVFDTGM IENTFQTNSA EALTYVNWAS
121 MLNLLLTGVL PAVLLYR**TQI AYKPMGK**ELA YKALYIIANV LIIVVIAFFY MQNYLAFGR**N**
181 **NDEIKRYIVP TYFVGSAAKY INQHYLQKPL EYQKLGTDAK QVKPAGETKP RLMVLVVGET**
241 **ARAQNYQYYG YDRPTNVYTK EISPLIFSDV SSCGTATAVS LPCMFSR**MSR EDYDSR**AAKA**
301 **QDTAVDVLNH AGVKVQWFDN DSGCK**GVCDR **VENKLIALNA DPKLCDGQFC QDQVLLDELK**
361 **QALLQPNNGQ DRLIVLHIIG SHGPTYYLRY PDQYRRFTPD CQRSDIQNCS REQLTNTYDN**
421 **TILYTDYIVS QVVEQLKGRS IDYDTAMMYL SDHGESLGEK GMYLHGSPYA IAPSEQTRVP**
481 **WMAWFSKQFK QQLGLNDDCL AK**EAAAK**SVS HDNLFDTLLG LFDVSSKVYR PELDLIALCR**
541 **R**

73.75% Coverage

Fig. S4 Biochemical characterization of NMCR-1
A. CD spectrum of NMCR-1 protein
B. ICP-MS profile of NMCR-1 protein
C. MS identity of NMCR-1 protein

The polypeptides matched are given in bold letters.

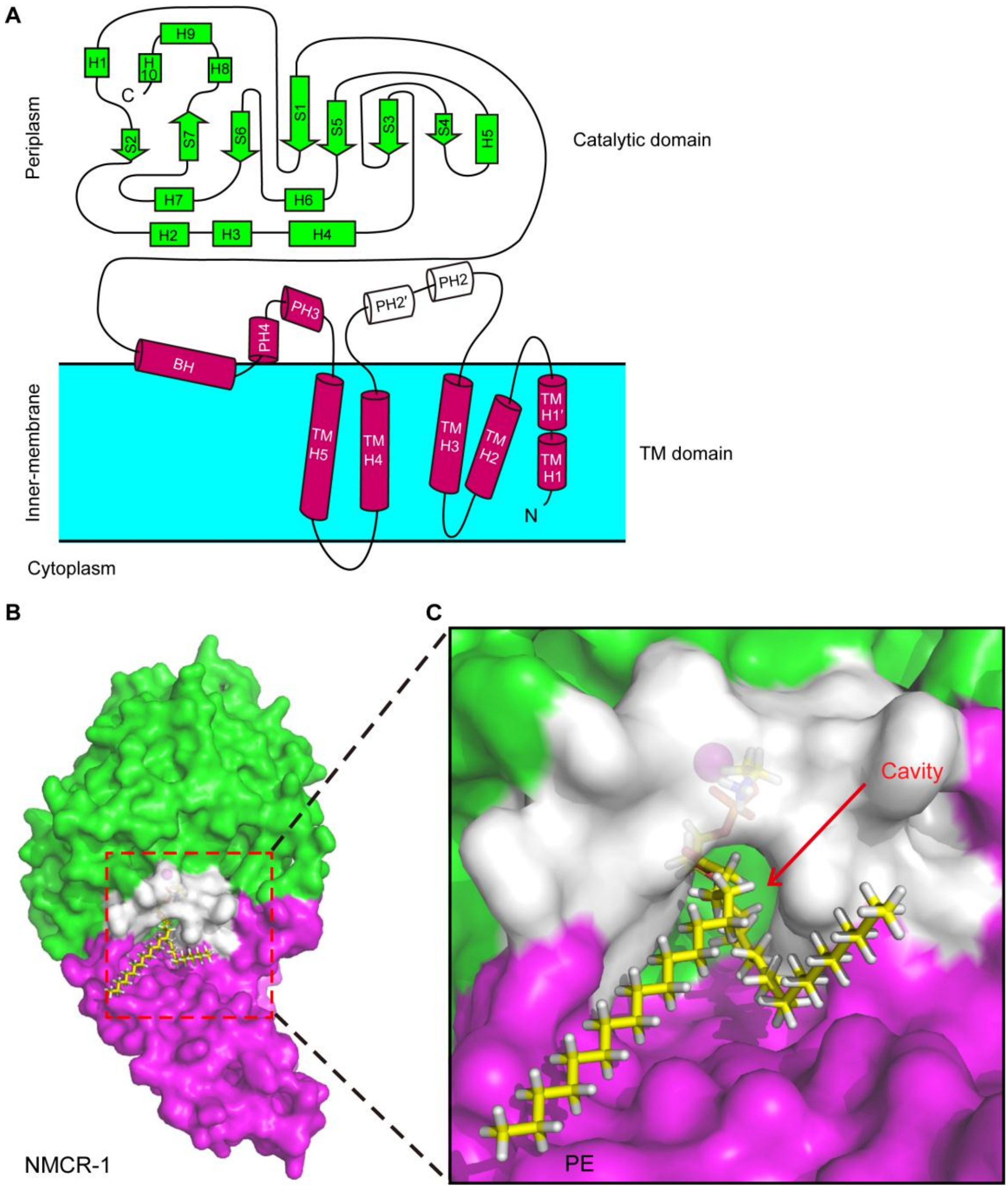

Fig. S5 Structural characterization of NMCR-1

A. Topological illustration of NMCR-1

B. Ribbon architecture of the modelled structure of NMCR-1 featuring with the cavity for the entry of PE lipid substrate

C. Enlarged view of the PE lipid substrate-recognizable cavity in NMCR-1

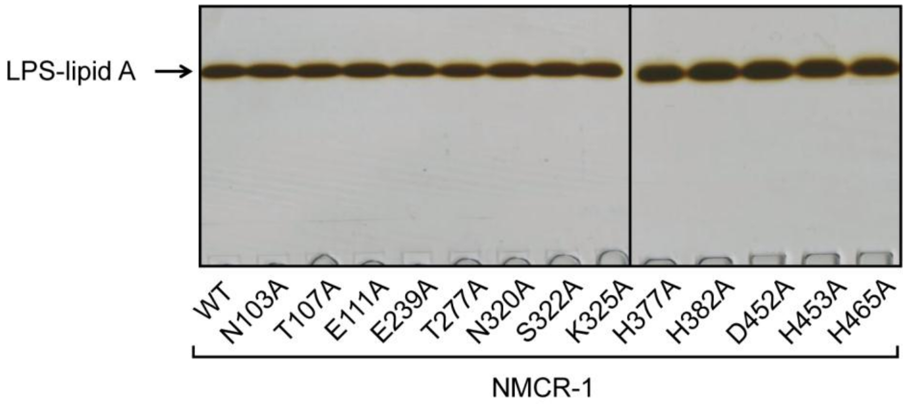

Fig. S6 Silver-staining analyses of LPS-lipid A species isolated from the *E. coli* expressing *nmcr-1* and different point-mutants

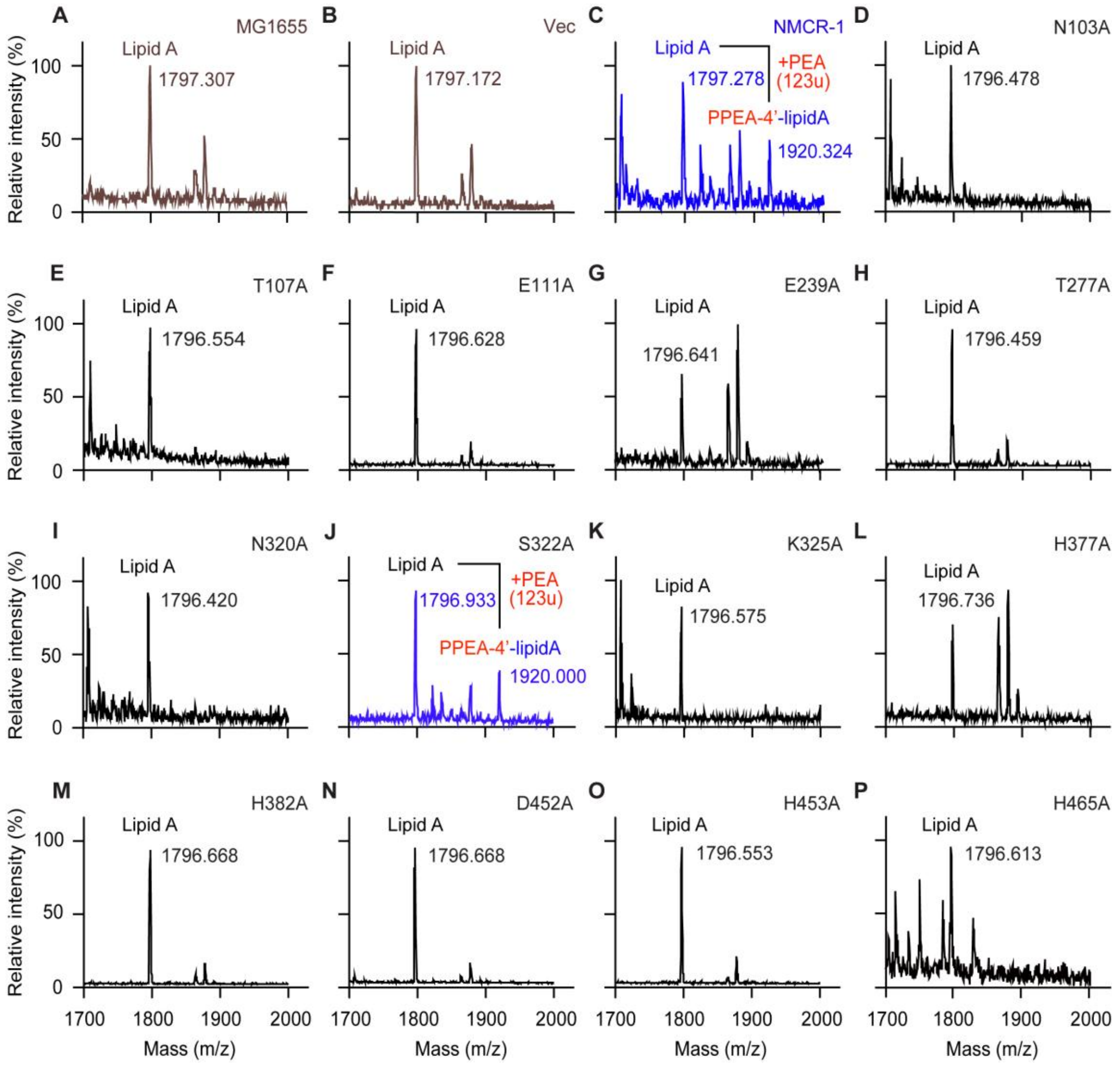

Fig. S7 MALDI-TOF MS evidence for physiological roles of the PE lipid substrate-interactive cavity of NMCR-1 in chemical modification of the LPS-lipid A in *E. coli*

A. MS spectrum of the LPS-lipid A species isolated from the colistin-susceptible strain *E. coli* MG1655 alone
B. MS profile of the LPS-lipid A species of the *E. coli* MG1655 strain containing the empty vector pBAD24
C. NMCR-1 modifies lipid A (m/z, 1796.992), giving the PPEA-4'-lipid A (m/z, 1920.145)

Five mutations of zinc-surrounded sites in NMCR-1 [namely E239A (**G**), T277A (**H**)], H377A (**L**), D452A (**N**), and H453A (**O**)] are inactive in the transferring of PEA to the suggestive 4'-phosphate position of lipid A species.

The point-mutant of S322A (**J**) in NMCR-1 retained partial activity in catalyzing the chemical decoration of lipid A moieties, and rest of mutants are

non-functional, including N103A (**D**), T107 (**E**), E111A (**F**), N320A (**I**), K325A (**K**), H382A (**M**), and H465A (**P**)], respectively.

Of note, the MS peak of lipid A species appears at m/z of 1796.404~1797.133. Expression of functional (and/or partial active) versions of *nmcr-1* in *E. coli* results in the presence of its modified form PPEA-4'-lipid A at m/z of 1919.488~1920.145.

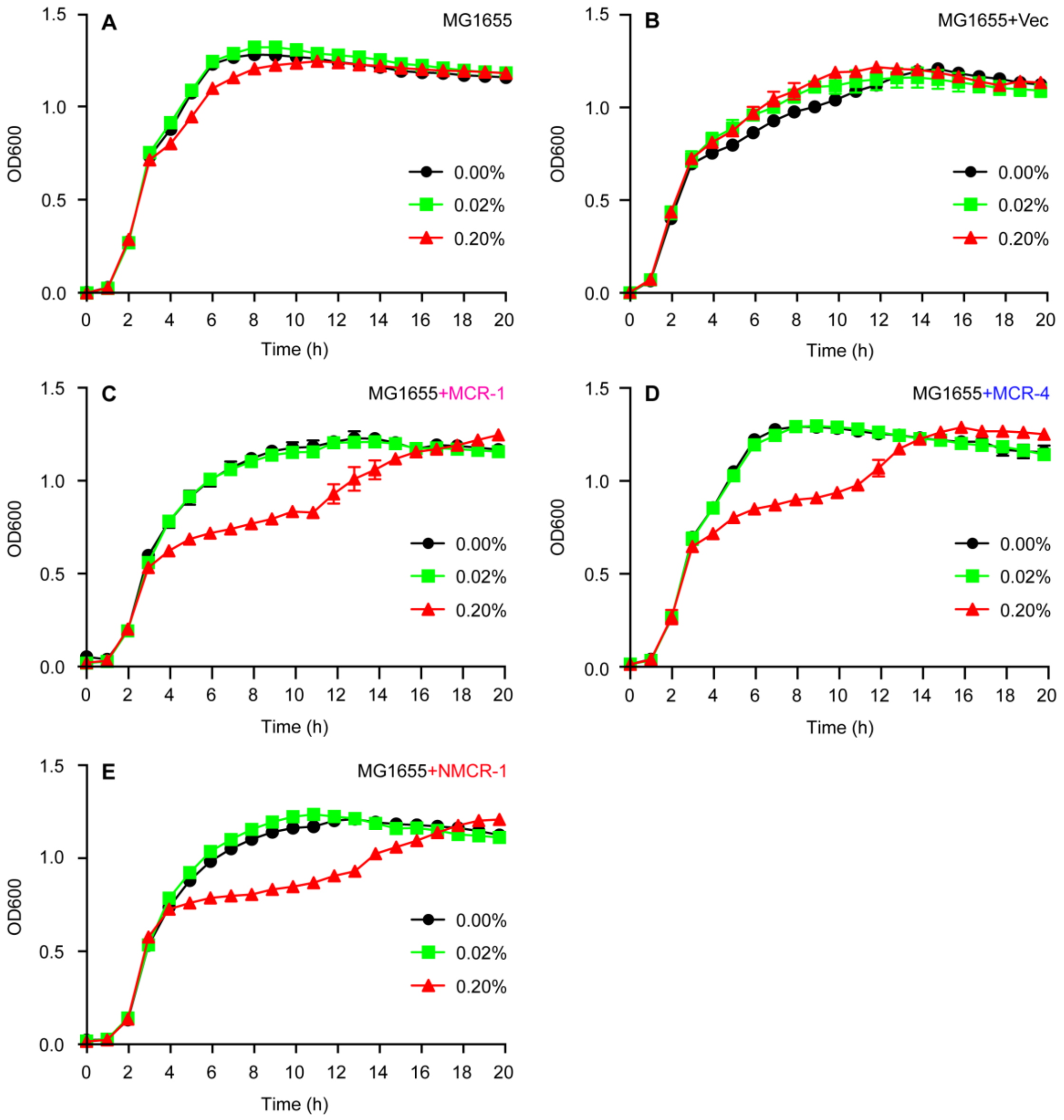

Fig. S8 Impact of NMCR-1 expression on bacterial growth

A. Growth curve of the *E. coli* MG1655 in LB medium at varied levels of colistin

B. Growth curve of the *E. coli* MG1655 carrying the empty vector pBAD24-8xHis in LB medium supplemented with different levels of colistin

C. Growth curve of the *E. coli* MG1655 in LB medium with colistin-induced expression of *mcr-1*

D. Expression of *mcr-4* shapes the growth curve of the *E. coli* MG1655 in LB medium

E. Inhibition on bacterial growth of the *E. coli* MG1655 in LB medium by the colistin-activated expression of *nmcr-1*

A representative result is given from three independent trials

Supplementary References

1. Q. Meng, J. Yin, M. Jin, H. Gao, Distinct nitrite and nitric oxide physiologies in *Escherichia coli* and *Shewanella oneidensis*. *Appl Environ Microbiol* **84**, e00559-00518 (2018); published online EpubJun 15 (10.1128/AEM.00559-18).
2. Y. Y. Liu, C. E. Chandler, L. M. Leung, C. L. McElheny, R. T. Mettus, R. M. Q. Shanks, J. H. Liu, D. R. Goodlett, R. K. Ernst, Y. Doi, Structural modification of lipopolysaccharide conferred by *mcr-1* in gram-negative ESKAPE pathogens. *Antimicrobial agents and chemotherapy* **61**, e00580-00517 (2017); published online EpubJun (10.1128/AAC.00580-17).
3. M. Biasini, S. Bienert, A. Waterhouse, K. Arnold, G. Studer, T. Schmidt, F. Kiefer, T. Gallo Cassarino, M. Bertoni, L. Bordoli, T. Schwede, SWISS-MODEL: modelling protein tertiary and quaternary structure using evolutionary information. *Nucleic acids research* **42**, W252-258 (2014); published online EpubJul (10.1093/nar/gku340).
4. A. Anandan, G. L. Evans, K. Condic-Jurkic, M. L. O'Mara, C. M. John, N. J. Phillips, G. A. Jarvis, S. S. Wills, K. A. Stubbs, I. Moraes, C. M. Kahler, A. Vrielink, Structure of a lipid A phosphoethanolamine transferase suggests how conformational changes govern substrate binding. *Proceedings of the National Academy of Sciences of the United States of America* **114**, 2218-2223 (2017); published online EpubFeb 28 (10.1073/pnas.1612927114).
5. P. Benkert, M. Kunzli, T. Schwede, QMEAN server for protein model quality estimation. *Nucleic acids research* **37**, W510-514 (2009); published online EpubJul (10.1093/nar/gkp322).
6. W. J. Allen, T. E. Balius, S. Mukherjee, S. R. Brozell, D. T. Moustakas, P. T. Lang, D. A. Case, I. D. Kuntz, R. C. Rizzo, DOCK 6: Impact of new features and current docking performance. *J Comput Chem* **36**, 1132-1156 (2015); published online EpubJun 05 (10.1002/jcc.23905).
7. J. J. Irwin, T. Sterling, M. M. Mysinger, E. S. Bolstad, R. G. Coleman, ZINC: a free tool to discover chemistry for biology. *J Chem Inf Model* **52**, 1757-1768 (2012); published online EpubJul 23 (10.1021/ci3001277).
8. E. F. Pettersen, T. D. Goddard, C. C. Huang, G. S. Couch, D. M. Greenblatt, E. C. Meng, T. E. Ferrin, UCSF Chimera--a visualization system for exploratory research and analysis. *J Comput Chem* **25**, 1605-1612 (2004); published online EpubOct (10.1002/jcc.20084).
9. R. A. Laskowski, M. B. Swindells, LigPlot+: multiple ligand-protein interaction diagrams for drug discovery. *J Chem Inf Model* **51**, 2778-2786 (2011); published online EpubOct 24 (10.1021/ci200227u).
10. F. Sievers, D. G. Higgins, Clustal Omega, accurate alignment of very large numbers of sequences. *Methods in molecular biology* **1079**, 105-116 (2014)10.1007/978-1-62703-646-7_6).
11. Y. Zhai, J. Tchieu, M. H. Saier, Jr., A web-based Tree View (TV) program for the visualization of phylogenetic trees. *Journal of molecular microbiology and biotechnology* **4**, 69-70 (2002); published online EpubJan (
12. H. Ye, Y. Li, Z. Li, R. Gao, H. Zhang, R. Wen, G. F. Gao, Q. Hu, Y. Feng, Diversified *mcr-1*-harbouring plasmid reservoirs confer resistance to colistin in human gut microbiota. *mBio* **7**, e00177 (2016); published online EpubApr 5 (10.1128/mBio.00177-16).
13. R. Gao, Y. Hu, Z. Li, J. Sun, Q. Wang, J. Lin, H. Ye, F. Liu, S. Srinivas, D. Li, B. Zhu, Y. H. Liu, G. B. Tian, Y. Feng, Dissemination and mechanism for the MCR-1 colistin resistance. *PLoS pathogens* **12**, e1005957 (2016); published online EpubNov (10.1371/journal.ppat.1005957).
14. H. Zhang, Q. Luo, H. Gao, Y. Feng, A new regulatory mechanism for bacterial lipoic acid

synthesis. *Microbiology Open* **4**, 282-300 (2015); published online EpubApr (10.1002/mbo3.237).

15. H. Zhang, B. Zheng, R. Gao, Y. Feng, Binding of *Shewanella* FadR to the *fabA* fatty acid biosynthetic gene: implications for contraction of the *fad* regulon. *Protein Cell* **6**, 667-679 (2015); published online EpubSep (10.1007/s13238-015-0172-2).
